# SIGffRid: A tool to search for sigma factor binding sites in bacterial genomes using comparative approach and biologically driven statistics

**DOI:** 10.1186/1471-2105-9-73

**Published:** 2008-01-31

**Authors:** Fabrice Touzain, Sophie Schbath, Isabelle Debled-Rennesson, Bertrand Aigle, Gregory Kucherov, Pierre Leblond

**Affiliations:** 1 Laboratoire Lorrain de Recherche en Informatique et ses Applications, Campus Scientifique, B.P. 239, UMR CNRS-INPL-INRIA-Nancy 2-UHP 7503, 54506 Vandœuvre-lès-Nancy, France; 2 Unité Mathématique, Informatique et Génome, INRA, 78350 Jouy-en-Josas, France; 3 Laboratoire de Génétique et Microbiologie, UMR INRA 1128, IFR 110, Université Henri Poincaré, B.P. 239, 54506 Vandœuvre-lès-Nancy, France; 4 Laboratoire d'Informatique Fondamentale de Lille, UMR USTL-CNRS 8022, 59655 Villeneuve d'Ascq, France

## Abstract

**Background:**

Many programs have been developed to identify transcription factor binding sites. However, most of them are not able to infer two-word motifs with variable spacer lengths. This case is encountered for RNA polymerase Sigma (*σ*) Factor Binding Sites (SFBSs) usually composed of two boxes, called -35 and -10 in reference to the transcription initiation point. Our goal is to design an algorithm detecting SFBS by using combinational and statistical constraints deduced from biological observations.

**Results:**

We describe a new approach to identify SFBSs by comparing two related bacterial genomes. The method, named SIGffRid (SIGma Factor binding sites Finder using R'MES to select Input Data), performs a simultaneous analysis of pairs of promoter regions of orthologous genes. SIGffRid uses a prior identification of over-represented patterns in whole genomes as selection criteria for potential -35 and -10 boxes. These patterns are then grouped using pairs of short seeds (of which one is possibly gapped), allowing a variable-length spacer between them. Next, the motifs are extended guided by statistical considerations, a feature that ensures a selection of motifs with statistically relevant properties. We applied our method to the pair of related bacterial genomes of *Streptomyces coelicolor *and *Streptomyces avermitilis*. Cross-check with the well-defined SFBSs of the SigR regulon in *S. coelicolor *is detailed, validating the algorithm. SFBSs for HrdB and BldN were also found; and the results suggested some new targets for these *σ *factors. In addition, consensus motifs for BldD and new SFBSs binding sites were defined, overlapping previously proposed consensuses. Relevant tests were carried out also on bacteria with moderate GC content (i.e. *Escherichia coli*/*Salmonella typhimurium *and *Bacillus subtilis*/*Bacillus licheniformis *pairs). Motifs of house-keeping *σ *factors were found as well as other SFBSs such as that of SigW in *Bacillus *strains.

**Conclusion:**

We demonstrate that our approach combining statistical and biological criteria was successful to predict SFBSs. The method versatility autorizes the recognition of other kinds of two-box regulatory sites.

## Background

The identification of Transcription Factor Binding Sites (TFBSs) is a fundamental problem in the understanding of regulatory networks. A large number of software programs have been designed for the identification of TFBSs. Some of them have been compared in a recent survey [1] that shows the diversity of proposed solutions. Many algorithms are devoted to single motifs prediction [2-11]. They include genetic algorithm [10], expectation maximization or Gibb sampling methods [2,5,7], with incorporated phylogeny data [11], or other methods often based on multiple alignments [4,6] or statistical over-representation [12] and can identify some kinds of TFBSs, but these approaches are not adapted to regulatory binding sites composed of two boxes (a box refers to a conserved part of a signal modelled by a word).

Indeed, in bacterial RNA polymerase, an interchangeable subunit, the sigma (*σ*) factor, recognizes motifs usually composed of two boxes called -35 and -10 in reference to their location with respect to the transcription initiation point. For close *σ *factors in related bacterial species, the spacer length between the two boxes of the sigma factor binding sites (SFBSs) can vary slightly [13]. This characteristic, however, is not tackled by most of the existing methods, such as the popular MEME program [2].

Consider the methods dedicated to finding two-box motifs. Most of them can not take into account the variability of spacer length between the two boxes [14-21]. At least four approaches deal with this property. Smile [22], and the more efficient and recent RISO [23], can search for two-box motifs and allows for variable spacer lengths, but they require defining precisely structural constraints applied to the motif in order to avoid a high number of output motifs. In addition, they require the user to define the minimal proportion of input sequences owning the motif looked for. Using a quorum as small as 0.8% to obtain motifs concerned by at least 8 sequences in a set of 1000 sequences gives in a very high number of results. A quorum as high as 10% needs the input set of sequences to be previously selected by another way to ensure that at least 10% of the sequences share the motif we search for. A motif recognized by a *σ *factor but corresponding to a small number of SFBSs could not be found. In practice, such algorithm can only be applied to sets of promoter regions of known possibly co-regulated genes. Nevertheless, they infer the more general problem of defining TFBS in eukaryotic organisms.

Closer to prokaryotic considerations, Jacques *et al*. algorithm [24] does not need transcriptional data and uses the supposed enrichment of transcription factor binding sites in intergenic regions. But it requires a matrix that represents the genomic distribution of hexanucleotide pairs, deduced from a training set composed of experimentally verified promoters, often from other bacteria when little is known in the bacterium we are interested in. The advantage of this algorithm is the variability of the spacer between boxes authorized for a same candidate as SFBS consensus. Unfortunately, this approach can not determine which nucleotides are important within each box and can not define the contribution of a position in a given SFBS. This contribution is variable depending on the bacterium for a same SFBS (illustrated by the Figure 6 of a recent article related to structural basis for -35 element recognition [25]). Given motifs are quite long compared to the number of conserved letters in the known promoters of *S. coelicolor *for example. This last remark is also applicable to MITRA application [18], and the algorithms implemented by Vanet *et al*. (tested on *Helicobacter pylori *[26]) which define 12-letter motifs. Another method based on Gibbs Sampling algorithm (Bioprospector [17]) requires specification of the width of the motif for the entire run, whereas motif length of SFBSs seems to be quite variable (see the review article [27]).

An appropriate way to improve results is the footprinting method, and more generally phylogenetic approaches because of the relative conservation of regulatory elements across evolution process. Current comparative approaches need either distantly related species or more than two species [10]. In the first case, the number of shared regulatory motifs will tend to decrease (in parallel to the decrease of motif conservation). In the last one, the need of a high enough number of known closely related bacteria will limit the approach to well-studied families of bacteria.

We present an algorithm, named SIGffRid, for identifying SFBSs, taking into account the limitations reported above. SIGffRid uses a comparative approach to guide word comparisons and defines two-box motifs, whose spacer length can vary sligthly. This possible variation is an important characteristic of SFBS motifs [13,28-32] that we have to take into account in the detection process. By restricting the set of searched conserved boxes to over-represented words at its footprinting stage, SIGffRid allows a comparison of closely related species that are more likely to share common regulatory elements and does not need a great number of bacteria. This phylogenetic footprinting limits false positive rate. The following stages treat each bacterium separately in order to obtain their peculiar motifs.

Our algorithm extends short pairs of patterns shared by conserved pairs of selected words, adapting box widths, until the global motif obtained reach a significant over-representation in upstream regions. It does not fix a strongly constrained structure for final SFBS candidates.

Within the treated set of orthologue pairs, SIGffRid looks for two-word motifs conserved in upstream sequences. If at least eight of those motifs in the same bacterium share the same seven-letter pattern (called motif in the following explanations), it can be considered to be a putative SFBS. The program does not need additional transcriptional data, but can use them with improved performances, if provided. Moreover, SIGffRid's final motifs can be composed with only seven bases. Therefore, subtle motifs can be found by our algorithm.

Most of the characteristics of SFBS motifs (spacer length and variability, box length) exploited by SIGffRid are already described by Hertz *et al*. [33] but had been combined only once, in an algorithm [24] that defines a SFBS with 12 nucleotides while some of known would need only seven, as is used in SIGffRid.

Phylogenetic relationships, motif properties, and statistical characteristics of SFBSs are the only selection criteria currently retained by our algorithm.

## Results

### Properties of SFBSs: parameters for the program

The parameters of SIGffRid are correlated to the biological characteristics of the SFBSs:

• the related -35 and -10 boxes, 3 to 7 letters wide (default values in SIGffRid), are sufficiently conserved for a same *σ *factor to be detected (6 fixed letters in the two boxes, at least 2 fixed letters per box). This motivates our interest to group putative SFBSs by homology of pairs of words.

In practice, in many cases, only one of the two boxes is well defined (the aptly-named extended -10 element for instance [34]), a fact taken into account by the capability of our algorithm to obtain motifs with various structures,

• minimal and maximal spacer lengths between -35 and -10 boxes, taking into account the binding sites of all *σ *factors can vary in a wide range of values (from 14 to 20 nucleotides by default for *σ*^70 ^family in SIGffRid),

• spacer length between the two boxes can vary slightly for the same *σ *factor in the same bacterium and for two orthologous *σ *factors in two related bacteria, characteristics taken into account by using variable spacer (± 1 by default in the same bacteria, reinforced by Agarwal *et al*. experiments [35] in an actinomycete; ± 1 by default in two related bacteria),

• SFBSs are located upstream of CDSs, property used for defining our *a posteriori *statistical test,

• each of the -35 and -10 boxes is over-represented in the whole genome if we consider frequencies of their sub-words. At its footprinting stage, SIGffRid restricts its set of conserved words to those significantly over-represented.

### General description

The main program needs following input data:

• the GenBank files of bacterial species of interest (from NCBI database),

• the file giving orthologous relationships (from MBGD database [36] possibly with a user file defining a list of interesting genes in one of the bacteria, or a user file defining orthologous gene IDs).

For the sake of clarity, we describe step by step, globally, the broad lines of the algorithm before refining their description.

We know that SFBSs occurrences are rare in a genome, because useless occurrences of SFBSs can represent a handicap for the bacterium which has to overcome the pressure of selection. The higher number of SFBS-like sequences the bacterium has (in non regulatory regions), the higher is the risk that it is counter selected as suggested by a recent study on density of promoter-like sequences for *σ*^70 ^[37]. When a transcription factor diffuses in the cell volume (or along with the DNA helix), it has to recognize its binding sites. It can only detect something which is exceptional compared to every possible motifs present in the genome. Selection pressure contributes both to the motif rareness and its conservation. Accordingly, we hypothesized -35 and -10 motifs of SFBSs as exceptional motifs in the genome. This was verified in *S. coelicolor *where all known sites owned either over-represented boxes or over-represented sub-words of boxes (minimal width of 3 letters)(data not shown). The algorithm is summarized as below:

#### Restrict dictionary of searched boxes

The searched boxes are the words detected by R'MES [38] as significantly over-represented in the whole genome of the bacterium of our interest. We chose the whole genome model because it is expected to be further from SFBS model than upstream sequence model. Therefore, SFBS boxes are more unexpected in the whole genome model.

#### Support for SFBS search

Using another closely related bacterium, intergenic sequences of probable orthologous genes are extracted and grouped by pairs. We chose to extract sequences from position -349 (largest value) to 0 in reference to translation start site because most of SFBSs are found in this range of positions (as shown by studies of *Escherichia coli *[39] or *Streptomyces *[40] promoters). We fixed their minimal length to 30 nucleotides.

*Though some SFBSs can occur in coding sequences, we use only intergenic sequences, otherwise we would have word conservation related to coding sequences, and consequently a high number of false positives. Nevertheless, for a putative SFBS motif, every occurrences located in the -349 to 0 regions are given in final result*.

#### Defining pairs of orthologues

We use orthologous relationships based on MBGD database [36], and group pairs of promoter regions of orthologous according to families given in MBGD, to decrease the number of sequences treated simultaneously.

*Although, these "families" are used to split the set of promoter regions in functionally consistent subgroups, we cannot systematically infer co-regulation relationships*.

SIGffRid gives the possibility for the user to define a subset of genes of one bacterium, thus, pairs of promoter regions obtained from orthologous relationship, if existing.

#### Defining conserved pairs of words

Then pairs of conserved over-represented words with a compatible spacer for a SFBS are searched: for each pair of orthologous promoter regions, a list of SFBS candidates shared by the two bacteria is obtained.

*Here, we consider the over-representation of each box on the whole genome even if we search only those conserved in promoter regions. Final statistical test will consider over-representation of the complete motif in upstream sequences of coding sequences*.

#### Grouping conserved pairs of words

Further, these pairs of words are grouped according to pairs of sub-words they share satisfying a spacer constraint. For this purpose, we fix sub-word profiles, called *seeds*.

#### Motifs extensions

From this stage, we treat the sequences of each bacterium separately, in order to find close motifs which could have diverged.

Finally, an extension of the shared sub-words is carried out according to a probabilistic model. Each one-letter extension concerns only one position and is followed by the design of a regular expression describing the conserved extended area.

#### Final statistical tests on candidate motifs

A statistical test is led on this regular expression to find out if it is specific to upstream regions of CDSs.

**Figure 1:**
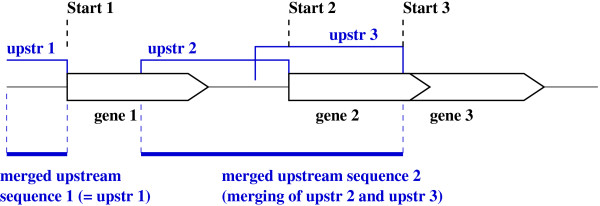
**Merging of upstream overlapping sequences on a same strand**. The final statistical test of motifs needs to count the number of occurrences in the upstream sequences. If genes overlap each other, their upstream sequences could overlap each other. We avoid to count twice the same motif occurrence by merging upstream overlapped sequences which are on a same strand.

Our statistical test is based on counting in two sets of sequences, and requires the using of:

○ *whole genome sequences*,

○ *lists of upstream sequences of CDS, merged if they overlap each other on a same strand, for each bacterium *(see Figure 1). *We count occurrences of possible SFBSs in these sets of sequences. Some SFBSs of a particular gene are known to be located in the upstream CDS. Therefore we use upstream sequences instead of intergenic upstream sequences to take every occurrence of SFBSs into account in the final statistical test*.

If the motif is considered as an interesting one, we then obtain annotations of genes located downstream the motif occurrences, and stop the process. Otherwise, it goes on recursively until an interesting motif is found.

We give a more detailed description of these techniques in the following paragraphs.

### Definition of searched words

R'MES [38] is a statistical software dedicated to finding words with exceptional frequencies in a sequence or a set of sequences. The exceptionality is evaluated by a statistical comparison between the observed counts and the ones expected under Markov models taking the sequence composition into account. A score of exceptionality is then calculated for each word. The study of -35 and -10 known boxes in *Streptomyces coelicolor *has shown that corresponding words, or sub-words they are composed of, get a high positive score, i.e. are significantly over-represented (data not shown). We have used this general property to restrict the number of searched words.

Here, we consider maximal order Markov models meaning that one takes the (*h*-1)-letter word composition of the sequences into account to find exceptional *h*-letter words. Since we consider words shorter than 8 bases in genomes longer than 8 Mb, i.e. very frequent words on average, scores are calculated using a Gaussian approximation of the count distribution [41]. Moreover, we analyze each word simultaneously with its reverse complement (considered like a word family in R'MES) because we run R'MES on the whole genome ; this is important as a mutation into a word on one strand leads to a mutation into its reverse complement on the other strand. Therefore, the frequency of a word is closely related to that of its reverse complement.

The scores of exceptionality produced by R'MES can be converted into approximate *p*-values. The *p*-value of an over-represented word is its probability to occur so many times in random sequences having the same short oligonucleotide composition than the observed sequence. More precisely, if *X *~ N(0, 1) then the approximate *p*-value is the probability for *X *to be greater than the observed score. Because of multiple testing, only words of length *h *with a *p*-value smaller than *α*/4^*h*^, with *α *= 5 *× *10^-3^, will be considered as exceptionally frequent; e.g. it corresponds to scores greater than 4.11 for *h *= 4 or than 4.71 for *h *= 6.

We applied this procedure to all words of length 3 ≤ *h *≤ 7 which gave us a set *W *of exceptionally frequent words on the alphabet A = {*a*, *c*, *g*, *t*}. These words were then searched in each pair of promoter regions of orthologues.

### Properties of candidate motifs as possible SFBSs

Let *sp*_*min *_and *sp*_*max *_be the minimal and maximal authorized spacers between -35 and -10 boxes (deduced from known SFBSs), and let *δ *be the spacer variation accepted in the SFBSs of two promoter regions.

**Figure 2:**
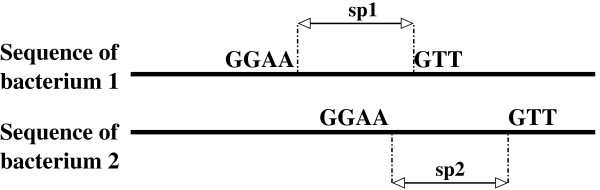
**Conservation of interesting words in promoter regions of orthologues**. We search for pairs of conserved significantly over-represented words with approximately the same spacer in the two promoter regions: *sp*2 - *sp*1 = *δ*, *δ *∈ {-1, 0, 1}.

Consider a triplet *C *= {*w*_1_, *w*_2_, {*s*_1*i*_, *s*_2*i*_}} corresponding to words *w*_1 _and *w*_2 _∈ *W *in promoter regions of orthologues *s*_1*i *_and *s*_2*i*_. *C *is said to be *interesting *if *w*_1 _and *w*_2 _occur in *s*_1*i *_and *s*_2*i *_with spacers *sp*1 and *sp*2 in [*sp*_*min*_, ...,*sp*_*max*_] respectively, such that -*δ *≤ *sp*2 - *sp*1 ≤ + *δ *(see Figure 2). For each pair of orthologous sequences, we keep only interesting triplets *C*. These are candidates as SFBS.

### Motif extensions

Next, we group interesting triplets according to pairs of seeds. We define a seed as a pattern *g *on the alphabet {*, #} where '*' can match with any character and '#' corresponds to an exact match.

For instance, from the seed *g *= ##*#, we get 3^4 ^searching motifs, or *keys*, on the alphabet A ∪ {*}:

$$\begin{array}{llll} \text{aa}*\text{a,} & \text{aa}*\text{c,} & \text{aa}*\text{g,} & \text{aa}*\text{t}\\ \text{ac}*\text{a,} & \text{ac}*\text{c,} & \text{ac}*\text{g,} & \text{ac}*\text{t}\\ \cdots & & & \\ \text{tt}*\text{a,} & \text{tt}*\text{c,} & \text{tt}*\text{g,} & \text{tt}*\text{t} \end{array}$$

**Figure 3:**
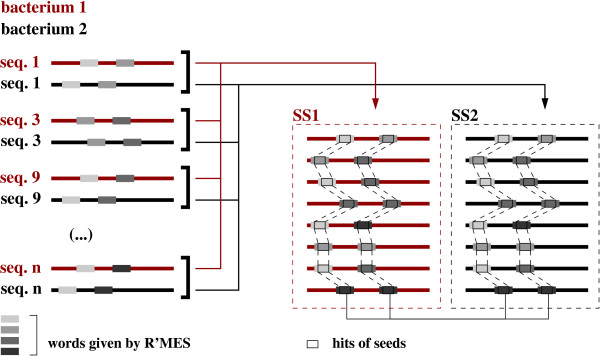
**Grouping of pairs of interesting words found in promoter regions according to pairs of hits**. From the conservation of pairs of words in the two bacteria (on the left of the Figure), we deduce the sets of sequences SS1 and SS2 – one for each bacterium – sharing a given pair of patterns.

Let *t*1 and *t*2 be two keys obtained from seeds *g*1 = ### and *g*2 = ### respectively, and let *d*_*t*1-*t*2 _be a spacer that separates *t*1 and *t*2 in a given *C*. By considering *SS*_1 _= ∪ *s*_1*i *_and *SS*_2 _= ∪ *s*_2*i *_(see Figure 3), a set C = {*t*1, *t*2, [*e*, ..., *e *+ *δ*], *SS*_1_, *SS*_2_} is deduced from all interesting *C *= {*w*_1_, *w*_2_, {*s*_1*i*_, *s*_2*i*_}} which verify, for a given integer *e*:

(*t*1 ⊂ *w*_1_) ∦ (*t*2 ⊂ *w*_2_) ∦ (*d*_*t*1-*t*2 _∈ [*e*, ..., *e *+ *δ*]).

For instance, using the key pair {*gaa*, *gtt*} obtained from seed pair {*g*1, *g*2} with *g*1 = *g*2 = ### and a spacer of *e *= 19 ± *δ*, the following pairs of words given by R'MES (in uppercase) for one bacterium will be grouped together (seeds are underlined):

gccgtgagggGAACact--atcggcgtagcgtGTTgagtcgcaa

caacaccgGGGAATagttc-accccgccccccgGTTttgggggat

tgatcccgcGGAATaggtcagctatggaccgtcGTTagcactcat

cggcagcCGGGAAtgggcgg-gccggtcgttcgGTTGccggg

We consider *λ *as the minimal number of distinct sequences (by default 8) involved in a candidate SFBS motif. We keep each set *SS*_1 _or *SS*_2 _only if it presents at least *λ *distinct sequences. Note that, for a given pair *t*1 and *t*2, we merge the sets C whose [*e*, ..., *e *+ *d*] intervals overlap each other.

A set *G *of possible seeds of length 3 ≤ *L *≤ 5 is fixed before the run. For grouping we use all non-redundant pairs of keys deduced from pairs of seeds {*g*1, *g*2} that verify

ℓ_*min *_≤ #(*g*1) + #(*g*2) ≤ ℓ_*max*_,

where ℓ_*min *_and ℓ_*max *_are respectively the minimal and maximal number of fixed authorized letters in the two seeds, and #(*g*) is the number of # in seed *g *(by default ℓ_*min *_= ℓ_*max *_= 6). To correspond with the usual form of SFBS motifs, we chose the set of seeds *G *= {###, ####, ##**#, #**##, #*#*#, #***#, #**#, #*#}. Motif extensions only concern seeds without '*' and composed of at least 3 letters (if one of the two seeds has gaps or is to short, only the other will be used for motif extension).

**Figure 4:**
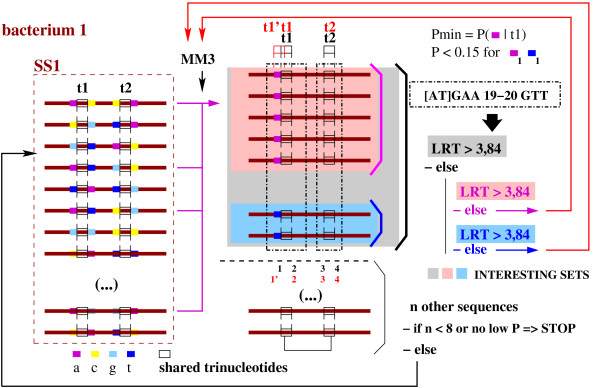
**Extension of shared trinucleotides, classifying of related promoter regions**. The set SS1 corresponds to *n *promoter regions of a given bacterium sharing a pair of given trinucleotides *t*1 and *t*2. We compute the probabilities to obtain the encountered letters at the positions neighbouring *t*1 and *t*2, considering our *n *sequences. We retain the position associated with the letter which has the lowest probability to be obtained as soon as observed in this set of *n *sequences. We group sequences according to the letters at this position which have a low probability to be obtained (with at least eight related sequences). They constitute new sets of sequences to be evaluated with LRT statistical test (see Section "*Computing a consensus motif and its statistical evaluation*"). "INTERESTING SETS" means sets of promoter regions whose shared motif is over-represented in merged usptream sequences.

For the sake of clarity, we will illustrate it for the case of pairs of trinucleotides (two seeds ###). Let *t*1 be the trinucleotide on the left which will be included in the -35 box of a potential SFBS and *t*2 be the trinucleotide on the right which will be included in the -10 box of the same potential SFBS. For each set *SS*_1 _and *SS*_2_, sequences are sorted according to the letters adjoining *t*1 and *t*2 (see Figure 4). We define the positions of letters as follows:

• position 1: immediately on the left of *t*1,

• position 2: immediately on the right of *t*1,

• position 3: immediately on the left of *t*2,

• position 4: immediately on the right of *t*2,

*Note that if t*1 *(respectively t*2*) corresponds to a gapped seed, positions 1 and 2 (respectively 3 and 4) are not used for extension and probability computations*.

Our statistical criteria uses the transition probabilities of a third-order Markov model adjusted on the whole genome. It means that probabilities are computed according to the three letters which come before/after the considered letter (depending on its position according to seed).

Let *n *be the number of sequences concerned, *t *be the trinucleotide to extend, and *j *∈ {1, 2} be the fixed subscript determining the treated sequences set. For a one letter extension on the right, we define:

$$Y_{i}^{r}(a)=\{\begin{array}{ll} 1 & \text{if the }i\text{-th sequence of }SS_{j}\text{ has}\\ & \text{the nucleotide }a\text{ at position }r,\\ & r\in \{2,4\},a\in A\\ 0 & \text{otherwise}. \end{array}$$

The number *N*^*r*^(*a*) of sequences having the nucleotide *a *at position *r*, i.e. $N^{r}(a)=\sum_{i=1}^{n}Y_{i}^{r}(a)$, follows the Binomial law B(*n*, *N*(*ta*)*/N*(*t*)), where *N*(·) is the counting function and *ta *the tetranucleotide formed with *t *followed by *a*. We can also compute the significance *p*^*r*^(*a*) of the observed number *x *of sequences with an *a *at position *r *:

$$p^{r}(a)=1-\sum_{y=0}^{x-1}\left(\begin{array}{c} n\\ y \end{array}\right){\left(\frac{N(ta)}{N(t)}\right)}^{y}{\left(1-\frac{N(ta)}{N(t)}\right)}^{n-y}.$$

For a one letter extension on the left, we apply the same principle: the number *N*^ℓ^(*a*) of sequences having *a *at position ℓ ∈ {1, 3} is distributed according to the Binomial B(*n*, *N*(*at*)/*N*(*t*)) and a *p*-value *p*^ℓ^(*a*) will be calculated.

We search for the position *k *∈ {1, 2, 3, 4} containing the minimal probability *p*_*k*_(*a*) over all *a *∈ A satisfying *N*_*k*_(*a*) ≥ *λ *. Let $A_{k}$ be the set of every letters *a *at position *k *which verify (*N*_*k*_(*a*) ≥ *λ*) ∦ (*p*_*k*_(*a*) ≤ 0.15). We group sequences according to each letter *a *∈ $A_{k}$ for the next steps (see Figure 4). A motif corresponding to this set of sequences is generated and evaluated (Section "*Computing a consensus motif and its statistical evaluation*").

• If it is considered to be an interesting one, we record the corresponding set of sequences as results,

• If the number of involved sequences becomes too low (<*λ*), the process is stopped,

• If the motif is not interesting, a new evaluation is done on each subset of sequences defined by letters from $A_{k}$.

○ if the evaluation is conclusive, we record the corresponding set of sequences as results,

○ otherwise the extension goes on every set of sequences defined by letters *a *from $A_{k}$, by replacing:

- *t*1 by *t*1' := *a*.*t*1[1].*t*1[2], if *k *= 1,

- *t*1 by *t*1' := *t*1[2].*t*1[3].*a*, if *k *= 2,

- *t*2 by *t*2' := *a*.*t*2[1].*t*2[2], if *k *= 3,

- *t*2 by *t*2' := *t*2[2].*t*2[3].*a*, if *k *= 4,

where . is the concatenation operator and *t*[*u*] stands for the *u*-th letter of trinucleotide *t*.

*Therefore, the extended area includes both t*1 *and t*1' *(respectively t*2 *and t*2'*) if k *= 1 *or k *= 2 *(respectively k *= 3 *or k *= 4*). In the first case, the extension process concerns the letters on the left of t*1 *and the right of t*1'*. In the second case, these are the letters on the left of t*2 *and on the right of t*2' *which are concerned*.

Other sequences are grouped and evaluated with the same criteria of probability for motif extension. Here, we take into account the fact that *σ *subunits of RNA polymerase are closely related if we consider regions of these proteins involved in -35 and -10 DNA binding (these regions are called 2.4 and 4.2 regions). Therefore, SFBSs might be so closely related that they differ only by one letter.

*Note: We verify that the sequence set cannot be split into several distinct subsets, each one corresponding to a spacer length with a narrower range of variation. If it is the case, we record each one of the results corresponding to subset, otherwise we record the global result*.

### Computing a consensus motif and its statistical evaluation

At each grouping step, a generic motif *m *is deduced corresponding to two words with a variable spacer. It is built by adding to (extended) trinucleotide pairs, bordering letters *a *∈ $A_{k}$ satisfying:

(*N*_*k*_(*a*) ≥ *λ*) ∦ (*p*_*k*_(*a*) ≤ 0.15)

where *p*_*k*_(*a*) is obtained from Equation (2) and *λ *is the minimal number of distinct sequences (by default 8) involved in a candidate SFBS motif.

The method evaluates the specificity of *m *for upstream sequences. The motif is then searched in the set *U *of upstream sequences of CDSs (we will call them merged sequences in this paper) by considering each strand separately (we merge sequences if they overlap each other on the same strand, see Figure 1). It means that we take into account the motif orientation when we search it in merged sequences. The number of occurrences is also computed on direct and reverse strands of the whole genome *G *(composed of |*G*| elements: genome, plasmids). We took into account plasmids because they usually contain genes with one particular interest like antibiotic resistance genes. We chose not to neglect regulatory elements located in plasmids.

Let ℓ_*U *_(respectively ℓ_*G*_) the length of *U *(resp. *G*) and *N*_*U *_(resp. *N*_*G*_) the number of occurrences of the motif *m *into *U *(resp. *G*). We then define the following ratio

$$R=\frac{N_{U}}{N_{G}}$$

that measures the specificity of the motif for merged sequences. To test the significance of *R*, we use the likelihood ratio test (LRT) [42]: the *LRT *statistics given below follows the chi-square distribution *χ*^2^(1) with one degree of freedom.

$$LRT=2\left[N_{U}\log\left(\frac{\frac{N_{U}}{N_{+}}}{\pi }\right)+N_{G}\log\left(\frac{\frac{N_{G}}{N_{+}}}{1-\pi }\right)\right]$$

where *N*_+ _= *N*_*U *_+ *N*_*G *_and $\pi =\frac{L_{U}\mu_{U}}{L_{U}\mu_{U}+L_{G}\mu_{G}}$ is the expected proportion of *m *occurrences in the merged sequences. *L*_*U *_and *L*_*G *_are the corrected lengths of sequences *U *and *G *(*L*_*U *_= ℓ_*U *_- (|*m*| × |*U*|), *L*_*G *_= 2 [ℓ_*G *_- (|*m*| × |*G*|)]) and *μ*_*U *_(resp. *μ*_*G*_) is the probability for the motif to occur in sequence *U *(resp. *G*) at a given position. *μ*_*U *_and *μ*_*G *_are calculated under the Bernoulli model (obtained from the sequence sets *U *and *G*) to take *U *and *G *nucleotide compositions into account. This is a crucial point because intergenic sequences are known to be richer in AT than other sequences in known bacteria whatever their GC letters proportion is [43].

*LRT *measures the difference of concentration of a given motif in two sets of sequences. The continuation or stop of the consensus motif extension -by sorting sequences- depends on *LRT*. A selection of the more interesting results is made according to the ratios *R *and *LRT*.

The relationship (*R *≥ *R*_*min*_) ∦ (*LRT *≥ *LRT*_*min*_) must be verified, with *LRT*_*min *_the quantile at 5% of the *χ*^2^(1) law and *R*_*min *_= $M\cdot \frac{\ell_{U}}{2\ell_{G}}$ the minimal threshold for specificity, where *M *corresponds to the minimal ratio between number of occurrences of SFBS supposed to be in merged sequences, and the number of occurrences in the whole genome in terms of number of occurrences (three by default). Considering that most SFBSs are in the upstream regions of CDSs, we suppose that sites which are located upstream are two times more represented in this set than in the whole genome (measurement of the density of the motif). This evidence makes SIGffRid to continue motif extension while motifs are not sufficiently specific to merged sequences (see Figure 4). Therefore, general elements quite frequent in upstream sequences but largely distributed on the whole genome are not in SIGffRid results.

### Visualization of the results

Each motif is displayed with all related gene identifiers, scores *R *and *LRT*. Two related files complete these results corresponding to all the occurrences found in the complete set of upstream sequences of the related bacterium (including plasmids), their positions according to the translation start point and the annotations of the involved genes. For validation, only cross checking with known biological pathway is necessary to ensure the coherence of related gene functions linked by the same regulation motif.

## Discussion

We ran SIGffRid on phylogenetically related bacterial species belonging to the same genus, *Streptomyces coelicolor *A3(2) and *Streptomyces avermitilis *MA-4680 [44,45]. These mycelial Gram-positive bacteria have large genomes (8,667,507 bp and 9,025,608 bp, respectively) and a high GC content (72.1% and 70.7%, respectively). Sixty nine percents of *S. avermitilis *genes have orthologues in *S. coelicolor *[45]. These bacteria present a complex regulatory network, as suggested by the high number of predicted *σ *factors (65 and 60, respectively), whose very few consensus regulatory binding sites are known. And approximately 12.3% of their genes are supposed to be regulators [44]. As proposed by Konstantinidis *et al*. [46], many regulation systems are expected to cross talk, because their genes share high sequence similarity (paralogous genes of expanded gene families), which suggests increased complexity in regulation as well.

In this context, defining SFBSs, and more generally TFBSs is a true challenge.

Genes of *S. coelicolor *and *S. avermitilis *were grouped according to functions defined in MBGD database [36] to reduce the memory and processor usage for large genomes. A total of 3,148 promoter pairs of orthologues were extracted, distributed in 15 functional categories (1,476 orthologous pairs), and the rest that could not be assigned to a function (1,672 pairs) were put in one single category. Spacer range was chosen to correspond to *σ*^70 ^family spacers (from 14 to 20). We used seeds {###, ####, ##**#, #**##, #*#*#, #***#, #**#, #*#} and the dictionary of exceptional words from *S. coelicolor *for the two bacteria (using *S. avermitilis *dictionary gave similar results, data not shown).

From our data set, 113 motifs (two words with a variable spacer) were obtained for *S. coelicolor *and 65 for *S. avermitilis*.

Additional file 1 summarizes most interesting results from SIGffRid (Table2_summary.pdf). The complete lists of putative binding sites, positions and sequences, and related gene functions for *S. coelicolor*, are also available on SIGffRid web page dedicated to results [47]. The SIGffRid web server can be found at [48]. To assign biological function to genes, we used the protein classification scheme available on Sanger Institute website [49] based on that originally created for *E. coli *in the EcoCyc database [50].

### Motifs and genes related to SigR binding site

To validate our approach, we looked for the presence of the SigR binding site among SIGffRid results. The regulon of SigR, a *σ *factor involved in the response to oxidative stress, is the largest described so far in *S. coelicolor*. Paget *et al*. show that SigR activates directly the response of at least 30 genes, and recognizes the motif GGAATN_18_GTT [51].

Two different motifs obtained with *S. coelicolor *overlapped the SFBS of SigR regulon (see Table 1). Among the 79 occurrences of the first motif GGAATN_16,19_GTT, 29 occured in the promoters previously described by Paget *et al*. [51]. The 30^*th*^, SCO3162 motif, was not found because it overlapped CDS. Rest of the 50 potential binding sites were cross referenced, with microarray data showing variation of genes transcription under thiol specific oxidative stress condition triggered by diamide containing medium (Paget MSB, personal communication). Four among them were differently expressed in the microarray data (SCO4956, SCO0569-0570, SCO4297, and SCO6061). Two of these motifs had a promoter with a 18 nt spacer (SCO4956, SCO0569-0570) and the other two had a 19 nt spacer (SCO4297, SCO6061). The unaltered expression of the genes related to the 46 other occurrences can be explained by either particular stress conditions inducing their transcription (not used in this microarray experiment) or by the fact that they are not real promoters.

**Table 1:** Summary of found motifs similar to known SigR SFBSs

***S. coelicolor *consensus: ***ggaatn*_18_*gtt *[51]						
SIGffRid motif	*R*	*LRT*		*N*_*U *_(1)	%_*U *_(2)	*N*_*U*∈*μ *_(3)

in *S. coelicolor*						
*ggaatn*_16,19_*gtt*	0.49	54.69		79	0.49	32
*gggaan*_18,20_*cgtt*	0.48	42.97		58	0.48	12

in *S. avermitilis*						
*ggaatn*_17,19_*gttg*	0.51	30.98		38	0.51	
*ggaatn*_17,18_*gttg*	0.60	30.59		31	0.60	
*gaatn*_17,18_*gttg*	0.44	25.36		40	0.45	

(1) *N*_*U *_is the number of occurrences found in merged sequences

(2) %_*U *_is the proportion of occurrences found in merged sequences (%_*U *_= *N*_*U*_*/N*_*G*_, where *N*_*G *_is the number of occurrences found in the whole genome on direct and reverse strand)

(3) *N*_*U*∈*μ *_is the number of occurrences in merged sequences related to a gene over-expressed in microarray data experiments under oxidative stress conditions, from Paget, personal communication

The second motif GGGAAN_18,20_CGTT corresponds to previously reported promoters likely regulated by the orthologue of SigR, named SigH, in another actinomycete *Mycobacterium tuberculosis *[51]. Twelve out of the 58 occurrences of this motif were related to differently expressed genes under oxidative conditions (SCO4039, SCO5805, SCO0888, and SCO6061 also reported above). Among these, eight were similar to the motif observed by Paget *et al*. [51]. Further, two occurrences (of the 12) shared the motif GGGAAGAN_16_CGTT (SCO0888, SCO4039), very close to the one previously deduced from SigH-dependent promoters in *M. tuberculosis *(GGGAACAN_16_CGTT [52]). One occurrence (SCO6061) also overlapped that of the first motif.

The Additional file 2 describes gene functions and proposed binding sites according to SIGffRid motifs similar to SigR binding site (Table3_SigR_motifs.pdf).

### Other putative binding sites of known sigma factors

Some motifs detected by SIGffRid correspond to proposed sigma factor binding sites. The motif CGTAAN_18,19_GTT matched the promoter of *bldM *(SCO4768 [53]), which is the sole known binding site for BldN. BldN is involved in morphological differenciation and recognizes the motif CGTAACN_16_CGTTGA.

The SIGffRid motif was found in 24 other regions upstream of coding sequences (see Additional file 3: Table4_BldN_motifs.pdf) suggesting new targets for the *σ *factor BldN.

HrdB, the major *σ *factor in *S. coelicolor *[54], has at least 12 known binding sites [54-62] of which six overlapped four SIGffRid motifs (TGACAN_17,20_AN_3_T, TTGAN_18,19_CTA, TTGACN_19,20_ANCNT, CNGN_18,21_TAGGCT). Five among the six remaining motifs, and the motif determined as HrdD binding site [59] (a close homologue of HrdB), were also close to the above four SIGffRid motifs. Approximately 390 genes would be concerned by those motifs.

### Other putative SFBSs

The SIGffRid motif, CNGN_14,16_AGTAA, could correspond to a SFBS consensus. Indeed, the motif CNGN_14,16_AGTAA is present in the promoter region of the *S. coelicolor bldB *gene and AGTAA has been proposed to be the -10 box of *bldB *[63]. The *bldB *gene encodes a 98 amino acids polypeptide involved in morphogenesis, antibiotic production, and catabolite control in *S.coelicolor *[63]. Interestingly, this motif is found in the DNA region preceding *bldKC*, belonging to a five gene cluster encoding an oligopeptide permease responsible for the import of an extracellular signal governing aerial mycelium formation in *S. coelicolor*.

Two SIGffRid results, TGTCAGTN_14,15_TnG and TGTCAGTN_14_TnG, found in both *S. coelicolor *and *S. avermitilis*, could correspond to DNA damage-inducible promoters. They are declinations of the *Streptomyces rimosus *UV-inducible *recA *promoter, given by Ahel *et al*. (TTGTCAGTGGCN_6_TAGggT [64]) and whose variation was proposed by Studholme *et al*. through a bioinformatic method [21]. Two additional motifs, TGTCAGTGN_9,12_ANG and TGTCAGTN_12,14_TNG, could be retrieved when the spacer length parameter range was made from 8 to 14. In *S. coelicolor*, 67 genes were featured by these motifs, and 39 of them were assigned to a function (see Additional file 4: Table5_recA_motifs.pdf). Several functional groups could be distinguished, the most significant being related to DNA repair (13–20 genes) and includes homologues of the *E. coli *genes *dinP*, *priA*, *radA*, *dinG*, *recQ*. This group also included DNA glycosylases (e.g. *ung*), excinuclease (e.g. *uvrB SC*), and polymerase I genes. Another set of genes was related to DNA replication (e.g. *dnaE*, *dnaN *encoding respectively *α *and *β *subunits of PolIII, and *recF*).

### TFBS motifs other than SFBSs

A SIGffRid motif, [TA]GTGAN_18,20_TN_2_C overlaps the BldD binding site whose consensus was proposed by Elliot *et al*. (AGTgAN_*m*_TCACc [65]). BldD is a key transcriptional regulator involved in morphological differentiation and antibiotic production in *S. coelicolor *[65]. This motif was found upstream of *bldG *(anti-sigma factor antagonist) and five *σ *factor encoding genes (including *HrdB*, and *whiG *which encodes an alternative sigma factor essential for sporulation [66]).

Another SIGffRid motif [TA]GTGAN_16,18_CNT overlapping the above motif was found upstream of seven *σ *factors, including *HrdD *and those found downstream of the first motif. We speculate that these motifs may be declinations of BldD binding site.

### Application to other bacterial genomes

The efficiency of SIGffRid was further tested onto pairs of related bacterial species with lower G+C genome contents (i.e. *Escherichia coli *K12, 50% and *Salmonella typhimurium *LT2, 52% on one hand, and *Bacillus subtilis *168, 43% and *Bacillus licheniformis *ATCC 14580 (DSM13), 46% on the other hand, [67-71]). Approximately 80% of the predicted *B. licheniformis *coding sequences have *B. subtilis *orthologues [70]. The phylogenetic relationships inferred from the 16S rDNA identities, 97.0% and 97.4% between the species of each pairs, was similar to those between the *Streptomyces *species (97.3%) previously used to develop the algorithm. In contrast to *Streptomyces *where functional gene categories were used to limit computational times and result quantities, the whole orthologue gene sets were used on *E. coli*/*S. typhimurium *and *B. subtilis*/*B. licheniformis *analyses.

Several motifs were proposed by SIGffRid for each pair. Among these motifs, some could describe the binding sites of the house keeping *σ *factors, *σ*^70 ^of *E. coli *and SigA of *B. subtilis*. Thus, for *B. subtilis*, the motifs *TTGAN*_18,19_*TATAAT *and *TTGACN*_18,20_*ATAAT *for instance perfectly match the known consensus. Some other motifs describe SFBSs for alternative *σ *factors such as SigW (*TGAAACN*_16,17_*CGTA *[72]) which is implied in stress response in *B. subtilis*. SIGffRID extends the -10 motif by one nucleotide to give *TGAAACN*_16,17_*CGTAT*. For *B. licheniformis*, the motif proposed match exactly the SigW consensus of *B. subtilis *described in the litterature. The data and additional motifs are detailed in Additional file 5 (Table6_eco_stm_bsu_bld.pdf).

## Conclusion

Our algorithm proved to be relevant in finding different SFBSs and TFBSs, and can be applied to any bacterial species because it only uses general properties. SIGffRid is particularly suited to the detection of SFBSs with a high number of occurrences (those of house-keeping *σ *factors, e.g. SigA in *B. subtilis*) or with a small number of well-conserved occurrences (those of some alternative *σ *factors, e.g. BldN or SigR binding sites in *S. coelicolor*).

We combine the knowledge of footprinting, constraints of motif structures, phylogeny and statistical models to ameliorate motif characteristics in TFBSs prediction.

Beyond phylogenetic footprinting, some features specific to our method take better into account the variations of the same SFBS in two closely related bacteria. The first being the extension of shared pairs of seeds applied separately in each bacterium. We eventually obtain different variations of the same SFBS in two related bacteria, where the differences concern boxes and/or spacer lengths. Another is its capabilities to group putative sites of the same transcription factor using probabilities. By analysing possible regulons found by SIGffRid, we have shown that regulatory networks could be deduced from annotations, in addition to consensus motifs. Finally, it features an independant statistical test to evaluate the pertinence of the motif. Based on a biological hypothesis, it has the advantage of allowing SIGffRid to be applicable on any subset of sequences (e.g. list of genes obtained from microarray data). Though SIGffRid can be improved by refining probabilistic models used for motif extension and statistical evaluation, it clearly infers motifs close to known consensuses of TFBS.

The nucleotidic motif is probably only one aspect of the SFBS recognition, but is a necessary first bioinformatic step for its prediction. It would be undoubtedly complicated to account for the large number of parameters implicated in DNA recognition by *σ *factors in all potential promoter regions.

## Authors' contributions

FT designed the combinatorial algorithm, with assistance from GK and IDR, and developed the SIGffRid application. SS developed the statistical methodology and wrote the statistical parts of the paper. FT wrote the biological parts of the paper, with assistance from PL and BA, and the computational parts, with assistance from GK and IDR. Results were interpreted by FT, with assistance from PL and BA. All authors read and approved the final manuscript.

## Supplementary Material

Additional file 1 **Summary of results for Streptomyces**. Summary of results. Interesting motifs given by SIGffRid when applied on *S. coelicolor *and *S. avermitilis *and comparison with known *σ *factor binding sites. Are given motifs whose occurrences overlap known SFBS (known motif is given in front of the name of the concerned Sigma factor). Click here for file

Additional file 2 **SIGffRid motifs related to SigR**. SIGffRid predictions related to the SigR target sequence in *S. coelicolor*. Gene functions and putative binding sites for SigR *σ *factor or its homologue(s). It shows overlaps of binding sites of the various motif declinations for SigR binding site. Click here for file

Additional file 3 **SIGffRid motifs related to BldN**. BldN related motif. Gene functions and putative binding sites for BldN *σ *factor in *S. coelicolor*. Click here for file

Additional file 4 **SIGffRid motifs possibly related to recA promoter motif**. Interesting motif related to *recA *promoter motif. Gene functions and putative regulatory binding sites for DNA-damage related motifs (*recA *promoter) in *S. coelicolor*. Click here for file

Additional file 5 **SIGffRid motifs similar to known E. coli, S. typhimurium, B. subtilis, or B. licheniformis SFBSs**. SIGffRid results compared with known SFBS motifs in *E. coli*/*S. typhymurium *on one hand, and *B. subtilis*/*B. licheniformis *on the other hand. Interesting results obtained from *E. coli *K12/*S. typhimurium *LT2 and *B. subtilis *168/*B. licheniformis *ATCC 14580 pairs of bacterial genomes by using all pairs of orthologues. Click here for file
