# Supplementary material for: SIGffRid: A tool to search for sigma factor binding sites in bacterial genomes using comparative approach and biologically driven statistics
Source: BMC Bioinformatics. 2008 Jan 31;9:73. doi: 10.1186/1471-2105-9-73 (PMC2375139; doi:10.1186/1471-2105-9-73)
Supplement: Additional file 1 — Summary of results for Streptomyces. Summary of results. Interesting motifs given by SIGffRid when applied on S. coelicolor and S. avermitilis and comparison with known σ factor binding sites. Are given motifs whose occurrences overlap known SFBS (known motif is given in front of the name of the concerned Sigma factor). [file 1471-2105-9-73-S1.pdf]

- (dark grey) **σ factor: name [known/deduced consensus if available]**
- (light grey) **known promoters (when no consensus can be clearly deduced)**
- (white) **SIGffRid output motifs**

[m4] links SIGffRid motifs to known promoter(s) it overlap

Table 1: SIGffRid results compared with known SFBSs motif

Motifs are presented as follow: DNA_motif (R ratio, LRT likelyhood ratio test).
(for *S. coelicolor*: $R \geq 0.48$, $LRT \geq 3.84$; for *S. avermitilis*: $R \geq 0.44$, $LRT \geq 3.84$)

<table>
<tr><td colspan="2">σ factor: HrdB</td></tr>
<tr><td colspan="2">known HrdB-dependent promoters overlapped by SIGffRid output motifs</td></tr>
<tr><td colspan="2">$?n_?taggct$ (SCO0598 [m4], sigBp2) [1]
$ttgaccn_{18}tacggt$ (SCO1429 [m3], chiD) [2, 3]
$ttgacan_{18}cacctt$ (SCO5003 [m1][m2], chiA) [3]
$ttgaccn_{17}tattct$ (SCO5376 [m3], chiC) [2, 3]
$ttgacan_{17}caaatt$ (SCO5673 [m1], chiB) [3]
$ttgacan_{17}tagatt$ (SCOr09 [m1], rrnDp2) [4, 5]</td></tr>
<tr><td colspan="2">other HrdB promoters strongly similar to SIGffRid motifs</td></tr>
<tr><td colspan="2">$tgggcan_{18}taggat$ (SCO2026, gltBp1) [6]
$ttgagcn_{18}taggtt$ (SCO0561, furA) [7]
$ttgtcan_{18}tagcat$ (SCO3471) [8]
$ttgactggtn_{14}ttgagt$ (SCO7263, chiF) [3]
$ttgaacn_{18}tagtgt$ (SCO2082, ftsZ2p) [9]</td></tr>
<tr><td colspan="2">SIGffRid output motifs (spacer range: 14 to 20)</td></tr>
<tr><td>S. coelicolor</td><td>S. avermitilis</td></tr>
<tr><td>$tgacan_{17,20}an_3t$ (0.50, 116.11)[m1]</td><td></td></tr>
<tr><td>$ttgacn_{19,20}ancnt$ (0.61, 65.76)[m2]</td><td>$ttgacn_{18,19}tac$ (0.47, 28.69)</td></tr>
<tr><td>$ttgan_{18,19}cta$ (0.60, 62.49)[m3]</td><td>$ttgan_{18,19}cta$ (0.49, 43.35)</td></tr>
<tr><td></td><td>$ttgn_{18,20}ccta$ (0.47, 67.51)</td></tr>
<tr><td></td><td>$ttgn_{17,19}cata$ (0.47, 45.32)</td></tr>
<tr><td>$cngn_{18,21}taggct$ (0.67, 100.33)[m4]</td><td>$cngn_{18,22}taggct$ (0.62, 101.89)</td></tr>
</table>

<table>
<tr><td colspan="2">σ factor: SigR $ggaatn_{18}gtt$ [10]</td></tr>
<tr><td colspan="2">SIGffRid output motifs (spacer range: 14 to 20)</td></tr>
<tr><td>S. coelicolor</td><td>S. avermitilis</td></tr>
<tr><td>$ggaatn_{16,19}gtt$ (0.49, 54.69)</td><td>$ggaatn_{17,19}gttg$ (0.51, 30.98)</td></tr>
<tr><td>$gggaan_{18,20}cgtt$ (0.48, 42.97)</td><td></td></tr>
<tr><td></td><td>$gaatn_{17,18}gttg$ (0.44, 25.36)</td></tr>
</table>

<table>
<tr><td colspan="2">σ factor: BldN $cgtaacn_{16}cgttga$ (SCO4768) [11]</td></tr>
<tr><td colspan="2">SIGffRid output motifs (spacer range: 14 to 20)</td></tr>
<tr><td>S. coelicolor</td><td>S. avermitilis</td></tr>
<tr><td>$cgtaan_{18,19}gtt$ (0.57, 20.04)</td><td>$gtaacn_{17,18}gtt$ (0.52, 17.81)</td></tr>
</table>

σ **factor: ?**, motif similar *Bacillus subtilis* SigE binding site [12] $catattn_{14}cataca$	
**SIGffRid output motifs (spacer range: 14 to 20)**	
S. coelicolor	*S. avermitilis*
$catan_{15,17}tac$ (0.54, 35.40)	
σ **factor (?) involved in sporulation regulation**	
**SIGffRid output motifs (spacer range: 14 to 20)**	
S. coelicolor	*S. avermitilis*
$cngn_{14,16}agtaa$ (0.49, 67.26)	
$agtaan_{13,15}cng$ (0.48, 58.48)	

**DNA-damage inducible promoters motif** $ttgtcagtggcn_6tag{\scriptstyle gg}t$ [13]	
**SIGffRid output motifs (spacer range: 14 to 20)**	
S. coelicolor	*S. avermitilis*
$tgtcagtn_{14,15}tng$ (0.59, 37.76)	$tgtcagtn_{14,15}tng$ (0.49, 36.73)
$tgtcagtn_{14}tng$ (0.63, 36.63)	
**SIGffRid output motifs (spacer range: 8 to 14)**	
S. coelicolor	*S. avermitilis*
$tgtcagtgn_{9,12}ang$ (0.85, 62.24)	
$tgtcagtn_{12,14}tng$ (0.64, 43.08)	$tgtcagtn_{12,14}tng$ (0.48, 36.05)

**TFBS consensus for: BldD** $agt{\scriptstyle g}an_mtcac{\scriptstyle c}$ (SCO) [14]	
**SIGffRid output motifs (spacer range: 14 to 20)**	
S. coelicolor	*S. avermitilis*
$[ta]gtgan_{18,20}tn_2c$ (0.57, 269.95)	$[ta]gtgan_{18,20}tn_2c$ (0.48, 242.80)
$[ta]gtgan_{16,18}cnt$ (0.55, 290.18)	$[ta]gtgan_{15,19}gn_2t$ (0.47, 400.42)
$tgtgan_{18,20}tna$ (0.50, 69.54)	$tgtgan_{18,20}tna$ (0.49, 99.17)
$tnan_{16,18}tgtga$ (0.57, 101.85)	$tnan_{16,18}tgtga$ (0.46, 100.52)
$tgtgan_{17,18}tnt$ (0.55, 76.95)	
$tntn_{16,19}ctgtga$ (0.48, 35.58)	

**Other interesting SIGffRid output motifs (spacer range: 14 to 20)**	
S. coelicolor	*S. avermitilis*
$taan_{17,21}gtta$ (0.73, 27.68)	$taan_{17,21}gtta$ (0.48, 18.33)
$cgggn_{13,15}tta$ (0.48, 80.37)	
$cccgn_{14,15}gtaa$ (0.52, 45.87)	

References

[1] Cho Y, Lee E, Ahn BE, Roe JH: **SigB, an RNA polymerase sigma factor required for osmoprotection and proper differentiation of *Streptomyces coelicolor* A3(2)**. *Mol Microbiol* 2001, **42(1)**:205–214.

[2] Delic I, Robbins P, Westpheling J: **Direct repeat sequences are implicated in the regulation of two *Streptomyces* chitinase promoters that are subjet to carbon catabolite control**. *Proc Natl Acad Sci USA* 1992, **89**:1885–1889.

[3] Saito A, Ishizaka M, Francisco PJ, Fijii T, Miyashita K: **Transcriptional co-regulation of five chitinase genes scattered on the *Streptomyces coelicolor* A3(2) chromosome**. *Microbiology* 2000, **146**:2937–2946.

[4] Baylis H, Bibb M: **Transcriptional analysis of the 16S rRNA gene of the rrnD gene set of *Streptomyces coelicolor* A3(2)**. *Mol Microbiol* 1988, **2(5)**:569–579.

[5] Kang JG, Hahn MY, Ishihama A, Roe JH: **Identification of sigma factors for growth phase-related promoter selectivity of RNA polymerases from *Streptomyces coelicolor* A3(2)**. *Nucleic Acids Res* 1997, **25(13)**:2566–2573.

[6] Brown K, Wood S, Buttner M: **Isolation and characterization of the major vegetative RNA polymerase of *Streptomyces coelicolor* A3(2); renaturation of a sigma subunit using GroEL**. *Mol Microbiol* 1992, **6**:1133–1139.

[7] Hahn J, Oh S, Roe J: **Regulation of the furA and catC operon, encoding a ferric uptake regulator homologue and catalase-peroxidase, respectively, in *Streptomyces coelicolor* A3(2)**. *J Bacteriol* 2000, **182(13)**:3767–3774.

[8] Buttner M, Brown N: **Two promoters from the Streptomyces plasmid pIJ101 and their expression in *Escherichia coli***. *Gene* 1987, **51**(2-3):179–186.

[9] Flärdh K, Leibovitz E, Buttner M, Chater K: **Generation of a non-sporulating strain of *Streptomyces coelicolor* A3(2) by the manipulation of a developmentally controlled *ftsZ* promoter**. *Mol Microbiol* 2000, **38(4)**:737–749.

[10] Paget M, Molle V, Cohen G, Aharonowitz Y, Buttner M: **Defining the disulphide stress response in *Streptomyces coelicolor* A3(2): identification of the σ^R regulon**. *Mol Microbiol* 2001, **42(4)**:1007–1020.

[11] Bibb M, Molle V, Buttner M: **σ^{BldN}, an Extracytoplasmic Function RNA Polymerase Sigma Factor Required for Aerial Mycelium Formation in *Streptomyces coelicolor* A3(2)**. *J Bacteriol* 2000, **182(16)**:4606–4616.

[12] Roels S, Driks A, Losick R: **Characterization of *spoIVA*, a sporulation gene involved in coat morphogenesis in *Bacillus subtilis***. *J Bacteriol* 1992, **174**:575–585.

[13] Ahel I, Vujaklija D, Mikoc A, Gamulin V: **Transcriptional analysis of the recA gene in Streptomyces rimosus: identification of the new type of promoter**. *FEMS Microbiology Letters* 2002, **209**:133–137.

[14] Elliot M, Bibb M, Buttner M, Leskiw B: **BldD is a direct regulator of key developmental genes in *Streptomyces coelicolor* A3(2)**. *Mol Microbiol* 2001, **40(1)**:257–269.
