# Supplementary material for: SIGffRid: A tool to search for sigma factor binding sites in bacterial genomes using comparative approach and biologically driven statistics
Source: BMC Bioinformatics. 2008 Jan 31;9:73. doi: 10.1186/1471-2105-9-73 (PMC2375139; doi:10.1186/1471-2105-9-73)
Supplement: Additional file 2 — SIGffRid motifs related to SigR. SIGffRid predictions related to the SigR target sequence in S. coelicolor. Gene functions and putative binding sites for SigR σ factor or its homologue(s). It shows overlaps of binding sites of the various motif declinations for SigR binding site. [file 1471-2105-9-73-S2.pdf]

Table 3: SIGffRid predictions related to the SigR target sequence (classification of genes according to the classification scheme for *S. coelicolor* available on http://www.sanger.ac.uk/Projects/S_coelicolor/classwise.html).
(1) Motif identified upstream the CDS: $GGAATN_{\{16,19\}}GTT$
(2) Motif identified upstream the CDS: $GGGAAN_{\{18,20\}}CGTT$
(P) Direct targets of SigR previously reported in Paget *et al.* 2001
Note: genes sharing the same motif can have the mention "see GeneID page PageNr" instead of the motif, most of the time in case of operon organization (motif is given in front of first gene). Motifs can occur in CDS: we search for in upstream sequences of CDS, intergenic or not. Upstream sequences overlapping each other on the same strand (according to downstrem genes) are merged. In this case, it is difficult to affect a motif to a gene: motif is given at front of the more probable possibly regulated gene, and other overlapping downstream genes sharing the same motif have the mention "see GeneID page PageNr".

0.0.0	**Unknown function, no known homologs (1049)**		
(1)	SCO0073 SCJ11.02	hypothetical protein	**ggaat**tatcggattcgttacc**gtt**
(1 P)	SCO0882 SCM1.15	hypothetical protein SCM1.15.	**ggaat**gttcaaccgtgcggcgtg**gtt**
(1)	SCO1107 2SCG4.23c	hypothetical protein 2SCG4.23c	**ggaat**gtgatcattcggttac**gtt**
(1 2 P)	SCO1421 SC6D7.18c	hypothetical protein SC6D7.18c.	**gggaat**ctttaccgccgcccgga**cgtt**
(1 P)	SCO1995 SC7H2.09c	hypothetical protein	**ggaat**agcgggtccggcgcgtgc**gtt**
(1 P)	SCO2912 SCE19A.12c	hypothetical protein	**ggaat**cgatacgatgagcccatg**gtt**
(1)	SCO3442 SCE36.09	hypothetical protein	**ggaat**gcgcagaggtcctcaccgg**gtt**
(1)	SCO3451 SCE46.08	hypothetical protein SCE46.08	**ggaat**gccgcgtacgactggaggg**gtt**
(2)	SCO4039 2SCD60.05c	hypothetical protein 2SCD60.05c	**gggaa**gatcgcgcgccgcgccga**cgtt**
(1 2 P)	SCO5163 SCP8.26c	hypothetical protein SCP8.26c	**gggaat**gtttgcagagggcatcc**cgtt**
(2)	SCO5310 SC6G9.23	hypothetical protein	**gggaa**cgggccgccacgcgcgca**cgtt**
(1)	SCO5727 SC3C3.13	hypothetical protein SC3C3.13	**ggaat**agccgaggaaagcatcga**gtt**
(1 2)	SCO6059 SC9B1.06	hypothetical protein SC9B1.06	**gggaat**cagcggaatctcccgttc**cgtt**
(1)	SCO6593	hypothetical protein	**ggaat**acccgaattctgaccg**gtt**
(2)	SCO6840 SC3D9.08c	hypothetical protein SC3D9.08c	**gggaa**atctcccattccgcgagc**cgtt**
(2)	SCO6860 SC7F9.12c	hypothetical protein SC7F9.12c	**gggaa**acgccggttagccaaaccgg**cgtt**
0.0.2	**Conserved in organism other than Escherichia coli (1322)**		

(1)	SCO0917 SCM10.05	putative oxygenase	**ggaat**ctgtgcggcgtgcggc**gtt**
(1 2)	SCO1304 SCBAC36F5.15	conserved hypothetical protein	**gggaat**cccggcacgtcgccgtc**cgtt**
(2)	SCO1542 SCL2.32	hypothetical protein	**gggaa**cgaaggcgggccgaccgc**cgtt**
(2)	SCO1645 SCI41.28c	conserved hypothetical protein SCI41.28c	(see SCO1646 page 2)
(2)	SCO1646 SCI41.29c	hypothetical protein	**gggaa**ccatggagatggtccccgta**cgtt**
(1 2)	SCO1869 SCI39.16c	conserved hypothetical protein	**gggaat**gagaacggctgcttgaa**cgtt**
(1 P)	SCO1997 SC7H2.11c	conserved hypothetical protein	**ggaat**gccccaccccgcaagggt**gtt**
(1 P)	SCO2161 SC6G10.34c	conserved hypothetical protein	**ggaat**gaatccgcggtcccgccg**gtt**
(2)	SCO2368 SCC8A.26c (and SCO2368-2367-2366-2365 posible gene cluster)	conserved hypothetical protein	**gggaa**cccgtcactaggatcaact**cgtt**
(1 P)	SCO2634 SC8E4A.04c	conserved hypothetical protein SC8E4A.04c	**ggaat**gggcgggccggtcgttcg**gtt**
(1)	SCO2816 SCBAC17F8.07	conserved hypothetical protein	**ggaat**aaccggagacccccctcct**gtt**
(1 P)	SCO2849 SCE20.23	conserved hypothetical protein SCE20.23.	**ggaat**cagcggtgggtacccgtg**gtt**
(1 P)	SCO2911 SCE19A.11c	conserved hypothetical protein	(see SCO2912 page 1)
(1 P)	SCO3187 SCE22.04	conserved hypothetical protein SCE22.04	**ggaat**aggtcactatggaccgtc**gtt**
(2)	SCO4390 SCD10.22	conserved hypothetical protein SCD10.22	**gggaa**ctcggtgtgtgctgcatca**cgtt**
(2)	SCO4419 SC6F11.17c	conserved hypothetical protein	**gggaa**cagccgcccgcccggccga**cgtt**
(1 P)	SCO5465 SC3D11.22	conserved hypothetical protein	**ggaat**agcccgcccggcccggag**gtt**
(2)	SCO5487 SC2A11.21c	conserved hypothetical protein SC2A11.21c	**gggaa**ccggagcgcgcgagtatg**cgtt**
(1)	SCO5533 SC1C2.14c	conserved hypothetical protein SC1C2.14c	**ggaat**aggtctagagtcgagctc**gtt**
(2,1 P)	SCO5754 SC7C7.09 and cluster of genes SCO5755-5756-5757-5758-5759	conserved hypothetical protein SC7C7.09, suggested *cinA*	**gggaa**ctcgggatcaccgtcctg**cgtt**<br>**gggaat**cgccgaacgcagagcgg**cgtt**
(1 2 P)	SCO5796 SC4H2.17	conserved hypothetical protein SC4H2.17, suggested *hflX*.	**gggaat**ctccccggtggttcgca**cgtt**
(1)	SCO6523 SC5C7.08	conserved hypothetical protein SC5C7.08	**ggaat**gcctcctgtctccggaccc**gtt**
(2)	SCO6776 SC6A5.25	conserved hypothetical protein	**gggaa**cacggatgtcacatccgt**cgtt**
(2)	SCO7294 SC5F8.04c	conserved hypothetical protein	**gggaa**ccccggccgcccggcgga**cgtt**

(2)	SCO7481 SCBAC17A6.14c	conserved hypothetical protein	**gggaa**ccctgtggttcccggccg**cgtt**
1.2.1	**Chromosome replication (8)**		
(1)	SCO3798 SCAC2.06c (SCO3798-3797 possible gene cluster)	possible chromosome condensation protein	**ggaat**gacctccgggctttcgtg**gtt**
1.3.1	**Chaperones (15)**		
(1)	SCO1648 arc SCI41.31c (SCO1648-1647-1646-1645-1644-1643 possible gene cluster)	AAA ATPase, Identical to *Streptomyces coelicolor* ARC	**ggaat**ccggccagctctgacgggt**gtt**
1.5.0	**Transport/binding proteins (520)**		
(1)	SCO1806 SCI33.05c	putative ABC transporter ATP-binding protein	**ggaat**accactctccgggcag**gtt**
(2)	SCO1903 SCI7.21	putative transport associated protein	**gggaa**cggtaaaacgccctcact**cgtt**
(1)	SCO1974 SC3C9.09	pseudogene, putative metal-transport protein	**ggaat**cccccgtttccacaggccc**gtt**
(1)	SCO2254 SC1G2.16c (SCO2254-2253 -2254 possible gene cluster)	putative transmembrane efflux protein	**ggaat**acccgccctgagagtcttt**gtt**
(1)	SCO2310 SCC53.01 SCC30.18 (SCO2310-2311 -2312-2313 possible gene cluster)	putative integral membrane efflux protein	**ggaat**agttgccccggcgcacgag**gtt**
(1)	SCO2505 SCC121.08 (SCO2505-2506 -2507-2508 possible gene cluster)	putative ABC-transporter metal-binding	**ggaat**cgtccgcttgttgacaacg**gtt**
(2)	SCO6258 SCAH10.23	putative ABC transport system sugar permease with signature domains related to xylose	**gggaa**gcaaggccgagatcgaca**cgtt**
1.7.1	**Cell division (20)**		

(1)	SCO2620 SCC80.05c (SCO2620-2619-2618-2617 possible gene cluster) , **OU SCOt16**	putative cell division trigger factor	(see SCOt16 page 8)
(2)	SCO3404 SCE9.11c ftsH2	ATP-dependent protease	**gggaa**cccctcgggcccccgcgc**cgtt**
2.1.3	**Degradation of polysaccharides (92)**		
(1)	SCO3471 dagA SCE65.07c (SCO3471-3470 -3469 possible gene cluster)	extracellular agarase precursor	**ggaat**gatcgtgccacgtggg**cgtt**
2.1.4	**Degradation of proteins, peptides, glycoproteins (126)**		
(1 2 P)	SCO2643 pepN SC8E4A.13	aminopeptidase N	**gggaat**gggtcgtgccgtgtggg**cgtt**
(2)	SCO4609 SCD39.09	putative peptidase	**gggaa**ctggtggccctcgcctgg**cgtt**
2.2.3	**DNA - replication, repair, restr./modific'n (85)**		
(2)	SCO5805 nrdJ	ribonucleotide reductase	**gggaa**caatcggggcacttcaggg**cgtt**
2.2.9	**Protein modification (12)**		
(1)	SCO4956 2SCK31.16	putative peptide methionine sulfoxide	**ggaat**cgtcgggagccctcccgt**gtt**
2.2.10	**Proteins - translation and modification (36)**		
(1)	SCO5360 prfA 2SC6G5.04 (SCO5360-5361 -5362-5363 -5364-5365 -5366-5367 -5368-5369 -5370-5371 -5372-5373 -5374-5375 possible gene cluster)	peptide chain release factor 1 (RF-1)	(see SCO5359 page 6)
3.1.6	**Cysteine (4)**		
(1 P)	SCO2910 SCE19A.10c	putative cysteine synthase	(see SCO2912 page 1)
3.1.9	**Glycine (3)**		
(2)	SCO2019 SC7H2.33c	putative chorismate mutase	**gggaa**cccgtgctcgaccggtccgc**cgtt**
3.2.2	**Biotin (7)**		

(1)	SCO2630 SC8E4.05c	putative biotin synthase	**ggaat**gcgatcatccgaatacggc**gtt**
3.2.5	**Folic acid (9)**		
(1 P)	SCO3403 SCE9.10c folE	putative GTP cyclohydrolase I	**ggaat**gtatgccgcaccccccag**gtt**
3.2.13	**Riboflavin (7)**		
(2)	SCO2688 ribD SCC61A.09	riboflavin-specific deaminase	**gggaa**accagctccgttcgcacgg**cgtt**
3.2.14	**Thiamin (7)**		
(1 P)	SCO5178 2SC3B6.02	putative sulfurylase	**ggaat**gaaaaggccatggcagt**gtt**
3.2.15	**Thioredoxin, glutaredoxin, glutathione (8)**		
(1 P)<br>(1 2 P)	SCO0885 trxA3 SCM1.18<br>SCO5187 2SC3B6.11c	thioredoxin<br>putative glutaredoxin-like protein	**ggaat**acccaccaggtcggcagt**gtt**<br>**gggaat**ggtcgtgccgccacgga**cgtt**
3.3.1	**2'-Deoxyribonucleotide metabolism (2)**		
(1 2 P)	SCO3890 SCH24.12c trxB	thioredoxin reductase (NADPH)	**gggaat**gcgcgccccgtaggatc**cgtt**
3.3.11	**Nucleotide interconversions (25)**		
(1 P)	SCO1513 relA SCL2.03c	GTP pyrophosphokinase	**ggaat**ccggcgcccgccccaggc**gtt**
3.4.4	**Fatty acids (59)**		
(1)<br>(1 P)<br>(2)	SCO1428 acd<br>SCO3415 SCE9.22<br>SCO7131 SC4B10.32	acyl-CoA dehydrogenase<br>putative esterase<br>putative lipase	**ggaat**tctttgagtaagcggtc**gtt**<br>**ggaat**catattccttggaatacg**gtt**<br>**gggaa**cgacgagcgactactggt**cgtt**
3.6.1	**Fatty acid and phosphatidic acid biosynth (53)**		
(2)	SCO5979 StBAC16H6.14	putative enoyl-CoA hydratase	**gggaa**gtgatcgtgcacggcaggtc**cgtt**
3.7.1	**Purine ribonucleotide biosynthesis (21)**		
(1 P)	SCO4770 SCD63.02 guaB	inosine 5' monophosphate dehydrogenase	**ggaat**gagccacgtccacttccc**gtt**
3.8.0	**Secondary metabolism (165)**		
(2)<br>(1)<br>(1 P)	SCO4339 SCD12A.22c<br>SCO6276 SC1G7.02<br>SCO6759 SC6A5.08	putative reductase<br>putative secreted protein<br>putative phytoene synthase	**gggaa**gccacttgccgaactccgga**cgtt**<br>**ggaat**gcctgatgaccggccc**gtt**<br>**ggaat**cttgaagtcaatcggtgt**gtt**
3.8.1	**PKS (102)**		
(1)	SCO4109 SCD17.13 SCD17A.0	putative oxidoreductase putative	**ggaat**cccccgctccggcgcggtc**gtt**

(1)	SCO6284 SC1G7.10	putative decarboxylase	**ggaat**cccggccgaggaggccgg**gtt**
4.1.6	**Gram +ve membrane (836)**		
(2)	SCO1023 SCG20A.03	putative membrane protein	**gggaa**ccccgtcctgggtccgcg**cgtt**
(1)	SCO1704 SCI30A.25	possible integral membrane conserved	**ggaat**ccgccagcacgcaccc**gtt**
(1)	SCO2350 SCC8A.08	putative integral membrane protein	**ggaat**gttgaacttgcaagtactc**gtt**
(2)	SCO2970 SCE59.29	putative membrane protein	**gggaa**cgttcgcgtcgtcgcggg**cgtt**
(1 P)	SCO3083 SCE25.24c	putative integral membrane protein	**ggaat**cactgatcggcgtagcgt**gtt**
(1)	SCO4007 2SC10A7.11c	putative integral membrane protein	**ggaat**gcgggtgagactcctcgg**gtt**
(2)	SCO4812 SCD63A.23c	putative integral membrane protein	**gggaa**cgcggcctgtgcgtcggg**cgtt**
(2)	SCO5763 SC4H8.02	putative membrane protein SC4H8.02	**gggaa**ctcggaagcagcccgttcg**cgtt**
(2)	SCO5826 SC5B8.16	putative membrane protein	**gggaa**gaagagtcggggatcttcc**cgtt**
(1)	SCO7202 SC2H12.01 SC1D2.0	putative membrane protein. hypothetical	**ggaat**gacgacaccgtactctg**gtt**
4.1.7	**Gram +ve exported/lipoprotein (439)**		
(2)	SCO0888 SCM1.21	putative secreted protein with NADPH-dependent FMN reductase signature	**gggaa**gagaactggaccgcggtc**cgtt**
(2)	SCO1650 SCI41.33c	putative secreted protein	**gggaa**ctccaccacggcgtcgacg**cgtt**
(1)	SCO2457 SCC24.28c	putative lipoprotein	**ggaat**gcctggaccccgcgga**gtt**
(1)	SCO6197 SC2G5.18c	putative secreted protein	**ggaat**cattccttcgcatatctc**gtt**
(1)	SCO6198 SC2G5.19	putative secreted protein	**ggaat**gattccggtttgcatt**gtt**
4.2.2	**Ribosomal proteins - synthesis, modification (62)**		
(1)	SCO0569 SC5G5.01c SC8B3.0	putative 50S ribosomal protein L36 (fragment)	(see SCO0570 page 6)
(1)	SCO0570 rpmG2 SC5G5.02c	50S ribosomal protein L33	**ggaat**gccccggcggccgtcggg**gtt**
(1 P)	SCO5359 rpmE3 2SC6G5.03	50S ribosomal protein L31	**ggaat**agttcaccccgcccccccg**gtt**
5.1.2	**Phage-related functions and prophages (25)**		
(1)	SCO5618 SC2E1.35	hypothetical protein SC2E1.35	**ggaat**gggcgtcagcccgcct**gtt**

5.1.3	**Plasmid-related functions (27)**		
(1)	SCO3995 SCBAC25E3.32	partial putative replication initiator protein	**ggaat**gagcacttggcagtcatc**gtt**
5.1.4	**Transposon/insertion element-related functions (85)**		
(2)	SCO0022 SCJ4.03c SCO0023	putative IS element ATP-binding protein	**gggaa**atgggcggcctcaatccgc**cgtt**
(2)	SCO3714 SCH35.10	putative transposase	**gggaa**ggtcccggcccgcgccaagg**cgtt**
6.1.1	**Sensor kinase (85)**		
(2)	SCO1744 SCI11.33	putative two-component system sensor kinase	**gggaa**gcgggcgtcaacctcacgc**cgtt**
6.2.1	**sigma factor (66)**		
(2)	SCO0255 SCF20.01c SCJ9A.3	putative transcriptional regulator putative	**gggaa**aagccgtacccaacccccc**cgtt**
(1)	SCO0632 SCF56.16	putative RNA polymerase sigma factor	**ggaat**gagaatccgtttctggg**gtt**
(1 2 P)	SCO3202 hrdD SCE22.19c	RNA polymerase principal sigma factor	**gggaat**tctgtccggattccagt**cgtt**
(2)	SCO4452 SCD6.30c	putative RNA polymerase sigma factor	**gggaa**gccgccggtcaccgacgggc**cgtt**
(2)	SCO5147 SCP8.10	putative ECF-subfamily sigma factor	**gggaa**cccgcagatgggaccagt**cgtt**
(1 P)	SCO5216 sigR SC7E4.13	RNA polymerase sigma factor	**ggaat**gaccggcggacggccact**gtt**
6.3.3	**GntR (51)**		
(1)	SCO3810 SCGD3.11c	putative gntR-family transcriptional regulator	**ggaat**ggcgttcattgagttg**gtt**
6.3.8	**TetR (122)**		
(2)	SCO3207 SCE22.24c	putative tetR-family transcriptional regulator	**gggaa**ataaccggggatcgtttccc**cgtt**
(1)	SCO6265 scbR SCAH10.30c	gamma-butyrolactone binding protein	**ggaat**catgtgatgccgagct**gtt**
(2)	SCO6784 SC6A5.33c	putative regulatory protein	**gggaa**ggcactgattccgaggaga**cgtt**
6.3.11	**LuxR (GerR) (23)**		
(1)	SCO5065 SCBAC20F6.08c	putative transcriptional regulator	**ggaat**acggccgcggtcctgccac**gtt**
6.3.13	**ArsR (15)**		
(2)	SCO6830 SC4A9.07	putative ArsR-family transcriptional regulator	**gggaa**cacctggaccattcctccgt**cgtt**
6.5.0	**Others (237)**		
(2)	SCO2259 SCC75A.05c	putative multi-domain regulatory protein.	**gggaa**acgtctcgcgggcggcggt**cgtt**

(1)	SCO4091 SCD25.27	putative DNA-binding protein	**ggaat**gaaccgagaaggttcg**gtt**
(1 2)	SCO7140 SC9A4.02	putative DNA-binding protein	**gggaat**gacgcaccccggacacga**cgtt**
7.0.0	**Not classified (included putative assignments) (565)**		
(2)	SCO1142 2SCG38.35	putative oxidoreductase	**gggaa**gatcggggcccgcgacgc**cgtt**
(1)	SCO3296 SCE15.13c	putative oxidoreductase	**ggaat**aacgggtcgggggtcgcc**gtt**
(1)	SCO3452 SCE46.09c	putative methyltransferase	**ggaat**gacgacggccgcgcagcgg**gtt**
(1)	SCO4297 SCD95A.30	putative oxidoreductase	**ggaat**ggtccggcggggcgggccc**gtt**
(2)	SCO5809 SC4H2.30	putative hydrolase	**gggaa**cgccgagatgcggatgct**cgtt**
(1 2)	SCO6061 SC9B1.08	putative oxidoreductase	**gggaat**ggcgaaggccggtgatct**cgtt**
(1 P)	SCO6551 SC4B5.01c	putative oxidoreductase	**ggaat**gcgtccgggctcgtgatc**gtt**
(1)	SCO7784 SC5E9.32	putative oxidoreductase	**ggaat**ggactcggcatggccctg**gtt**
	**Not reported in classification scheme**		
(1 P)	SCOs02		**ggaat**aggctggcacgcgtccgt**gtt**
(1)	SCOt16		**ggaat**cgaacccgcgtagcca**gtt**
(2)	SCOt28		**gggaa**actgtgtagacttgccga**cgtt**
(2)	SCP1.130	hypothetical protein	**gggaa**catctggtcgacgtgtccc**cgtt**
(1)	SCP1.247c	hypothetical protein	(see SCP1.248c page 8)
(1)	SCP1.248c	hypothetical protein	**ggaat**gcttcggaaacccca**gtt**
