# Supplementary material for: SIGffRid: A tool to search for sigma factor binding sites in bacterial genomes using comparative approach and biologically driven statistics
Source: BMC Bioinformatics. 2008 Jan 31;9:73. doi: 10.1186/1471-2105-9-73 (PMC2375139; doi:10.1186/1471-2105-9-73)
Supplement: Additional file 3 — SIGffRid motifs related to BldN. BldN related motif. Gene functions and putative binding sites for BldN σ factor in S. coelicolor. [file 1471-2105-9-73-S3.pdf]

Table 4: Classification of *Streptomyces coelicolor* genes with a putative SFBS $CGTAAN_{\{18\,19\}}GTT$ in their upstream region

0.0.0	Unknown function, no known homologs (1049)		
	SCO3324 SCE68.22	hypothetical protein	**cgtaa**cccaagtgccgcgccgctc**gtt**
	SCO3374 SCE94.25	hypothetical proline-rich protein	**cgtaa**cccgagcgctctttcgact**gtt**
	SCO6909 SC1B2.15	hypothetical protein SC1B2.15	**cgtaa**ttcactaagtgacagtgcc**gtt**
0.0.2	Conserved in organism other than Escherichia coli (1322)		
	SCO4398 SCD10.30c	conserved hypothetical protein	**cgtaa**cccccggtgggcccgggag**tt**
	SCO4543 2SCD4.14c	hypothetical protein	**cgtaa**gcggtacagggacaggtgag**tt**
	SCO5581 SC7A1.25c	conserved hypothetical protein	**cgtaa**ccgccgctgcgtcggaccc**gtt**
1.5.0	Transport/binding proteins (520)		
	SCO2422 SCC42.03c	putative ABC-transporter ATP-binding protein	**cgtaa**cgtgccggtgcggtgctcg**gtt**
	SCO6483 SC9C7.19	putative efflux protein	**cgtaa**ctcgtcgcacttgttcgca**gtt**
2.1.3	Degradation of polysaccharides (92)		
	SCO2430 SCC24.01 SCC42.11	putative sugar hydrolase (fragment) (putative secreted protein) putative secreted beta-galactosidase (fragment)	**cgtaa**cccgggccacggcctacgg**gtt**
	SCO7019 aml	secreted alpha-amylase.	**cgtaa**gagatttctgcaactttcc**gtt**
2.2.1	Amino acyl tRNA syn; tRNA modific'n (34)		
	SCO1595 pheS	putative phenylalanyl-tRNA synthetase alpha chain	**cgtaa**tccgcgttccacagcccc**gtt**
2.2.4	Glycoprotein (11)		
	SCO2981 SCE50.09	putative glycosyl transferase	**cgtaa**ccaagcgggtccaccggcc**gtt**
2.2.10	Proteins - translation and modification (36)		
	SCO1528 SCL2.18	putative elongation factor	**cgtaa**aaaatgcacgcgcgtactt**gtt**
3.6.0	Fatty acid biosynthesis (3)		
	SCO6473 SC9C7.09c	crotonyl CoA reductase	**cgtaa**agatttcatctgagaagag**tt**
3.8.0	Secondary metabolism (165)		
	SCO5799 SC4H2.20	putative aminotransferase	**cgtaa**ccccgtgaacggtcctcg**gtt**

4.1.6	Gram +ve membrane (836)		
	SCO1540 SCL2.30c	putative membrane protein	cgtaactcttccggccgatggaccgtt
4.1.7	Gram +ve exported/lipoprotein (439)		
	SCO5202 2SC3B6.26	putative secreted protein	cgtaacccgccccccggcgtgagcgtt
5.1.2	Phage-related functions and prophages (25)		
	SCO5612 SC2E1.29	putative ATP binding protein	cgtaagcggctcctccgtctcctgtt
6.3.5	LacI (34)		
	SCO7014 SC1H10.03c	probable LacI-family transcriptional regulatory protein	cgtaacggggatcggcaacgactgtt
6.3.8	TetR (122)		
	SCO3367 SCE94.18	putative tetR-family regulator	cgtaactagccgggcgtcaagtaagtt
6.3.11	LuxR (GerR) (23)		
	SCO4768 SC6G4.46	putative two-component regulator	cgtaactctttcgagtgaccatcgtt
	Not known according to classification		
	SCP1.28c<br>SCP1.167<br>SCP1.230 mmyB<br>SCP1.326		cgtaagccggaggaagaggacgtagtt<br>cgtaacagcgcggtggacatcaccgtt<br>cgtaagccccggtttaactctccgtt<br>cgtaagccggaggaagaggacgtagtt
