# Supplementary material for: SIGffRid: A tool to search for sigma factor binding sites in bacterial genomes using comparative approach and biologically driven statistics
Source: BMC Bioinformatics. 2008 Jan 31;9:73. doi: 10.1186/1471-2105-9-73 (PMC2375139; doi:10.1186/1471-2105-9-73)
Supplement: Additional file 4 — SIGffRid motifs possibly related to recA promoter motif. Interesting motif related to recA promoter motif. Gene functions and putative regulatory binding sites for DNA-damage related motifs (recA promoter) in S. coelicolor. [file 1471-2105-9-73-S4.pdf]

Table 5: Classification of *Streptomyces coelicolor* genes with a putative SFBS $TGTCAGTN_{\{14,15\}}TNG^{m4}$, $TGTCAGTGN_{\{9,12\}}ANG^{m5}$, and/or $TGTCAGTN_{\{12,14\}}TNG^{m6}$ in their upstream region (similar to *recA* promoter). Genes are grouped according to the classification scheme for *S. coelicolor* available on http://www.sanger.ac.uk/Projects/S_coelicolor/classwise.html.
Genes without ^{m4}, ^{m5} *or* ^{m6} *label own to a possible cluster (with previously cited gene) and are cited because they may be related to DNA damage.*
Therefore, those genes DO NOT correspond to the classification!!!!

0.0.0	Unknown function, no known homologs (1049)	
SCO0031^{m5} SCJ4.12c	hypothetical protein	tgtcagtgctcgttcatatg
SCO0268^{m4m5m6} SCF1.10	hypothetical protein	tgtcagtggcgtgggcaatggttg
SCO0558^{m4m5} SCF73.05c	hypothetical protein SCF73.05c	tgtcagtggcggatgcgaggcttcg
SCO0679^{m4m6} SCF91.39	hypothetical protein SCF91.39.	tgtcagtgccggaacacgtccttg
SCO1256^{m4m6} 2SCG18.03c	hypothetical protein 2SCG18.03c	tgtcagtggcggcgggtttactgg
SCO1291^{m5} SCBAC36F5.02	hypothetical protein SCBAC36F5.02	tgtcagtggtcgcctctacg
SCO1334^{m4m5m6} 2SCG61.16	hypothetical protein	tgtcagtgcttgcccctaggcttg
SCO2205^{m4m6} SC3H12.13c	hypothetical protein SC3H12.13c	tgtcagtggtgggcagtaaaatag
SCO2211^{m4m5m6} SC10B7.06	hypothetical protein SC10B7.06	tgtcagtggcccgtgcgagggtgg
SCO2738^{m4m6} SCC57A.09c	hypothetical protein SCC57A.09c.	tgtcagtccccaccggtaccgtcg
SCO2791^{m5m6} SCC105.22c	hypothetical protein SCC105.22c	tgtcagtggccaatccccgtagg
SCO2975^{m5} SCE50.03	hypothetical protein SCE50.03	tgtcagtggcaggtgcgaggatg
SCO3050^{m4m5m6} SCBAC19G2.05c	hypothetical protein	tgtcagtggtgggtgagacggtgg
SCO4611^{m5} SCD39.11	hypothetical protein SCD39.11	tgtcagtggcggcctgcatcatg
SCO6674^{m4m5m6} SC5A7.24c	hypothetical protein SC5A7.24c	tgtcagtggtggacggcaagctgg
SCO6735^{m4m5m6} SC5F2A.18	hypothetical protein	tgtcagtggtggcccgtacggtgg
SCO7634^{m4m6} SC10F4.07	hypothetical protein SC10F4.07	tgtcagtgggtgatggcagcctgg
SCO7723^{m5} SC8D11.14c	hypothetical protein SC8D11.14c	tgtcagtgggtcccggcagg
SCO7787^{m5} SC5E9.35c	hypothetical protein	tgtcagtgttcatggcggacg
0.0.2	Conserved in organism other than Escherichia coli (1322)	
SCO0180^{m6} SCJ1.29c	conserved hypothetical protein SCJ1.29c	tgtcagtgcccccgtcgccgtcg

SCO0563^{m5} SCF73.10c	conserved hypothetical protein SCF73.10c	tgtcagtgggacctcgtacg
SCO1653^{m5} SCI41.36	conserved hypothetical protein SCI41.36	tgtcagtgcccggccgtaagg
SCO1731^{m4m5m6} SCI11.20c	conserved hypothetical protein	tgtcagtggcggttgtcacg<br>tgtcagtggcgggtgccaccctgg
SCO2204^{m5} SC3H12.12	hypothetical protein SC3H12.12	tgtcagtggggtcgggcacgatg
SCO4113^{m4m6} SCD17A.05c	conserved hypothetical protein	tgtcagtggcgcagtgcactatcg
SCO4803^{m4m6} SCD63A.14	conserved hypothetical protein	tgtcagtggcgtggtgcaccgtgg
SCO6120^{m5} SC9B2.07	hypothetical protein SC9B2.07	tgtcagtggttactgcaaccatg
SCO7098^{m5} SC3A4.24c	conserved hypothetical protein SC3A4.24c.	tgtcagtggcaacaattgtcagg
1.2.1	Chromosome replication (8)	
SCO2064^{m4} SC4G6.33c dnaE	DNA polymerase III alpha chain	tgtcagtcccgaggcatagacttcg
SCO3878^{m4m6} dnaN SCH18.15c	DNA polymerase III, beta chain	tgtcagtggggtgcgtcagactgg
SCO3877	putative 6-phosphogluconate dehydrogenase	
SCO3876 recF SCH18.13c	DNA replication protein	
1.5.0	Transport/binding proteins (520)	
SCO1965^{m5} SCC54.25c	putative export associated protein	tgtcagtgcccctgcgtagg
SCO2373^{m4m6} tcmA SCC8A.31c SC	tetracenomycin C efflux protein. tetracenomycin C resistance	tgtcagttactgactcgtgaattgg
SCO4963^{m5} 2SCK31.23	putative ABC transporter ATP-binding protein	tgtcagtggtcgacgctaacg
SCO5451^{m4m5m6} SC3D11.08c	putative ABC transporter	tgtcagtggcaaaccgtaggctcg
SCO6805^{m5} SC1A2.14	putative integral membrane efflux protein.	tgtcagtgccccgtggcatg
1.6.1	Adaptations, atypical conditions (25)	
SCO4505^{m5} scoF2 SCD35.12c	**cold shock** protein	tgtcagtggctgccactagg
2.1.1	Degradation of DNA (13)	
SCO6341^{m5m6} SC3A7.09	putative exonuclease	tgtcagtgccgcccggtaggttg
2.2.3	DNA - replication, repair, restr./modific'n (85)	
SCO1343^{m5} ung 2SCG61.25c	uracil-DNA glycosylase (EC 3.2.2.-)	tgtcagtggtcggcggtagg
SCO1380^{m4m6} SC10A9.22c	putative DNA damage inducible protein, N-terminal region similar to SW:DINP_ECOLI (EMBL:D83536) Escherichia coli DNA-damage-inducible protein P DinP.	tgtcagtggcgtagggcagcatcg

SCO1475[m5] SCL6.32c	putative primosomal protein n', similar to SW:PRIA_ECOLI (EMBL:M33293) Escherichia coli primosomal protein n' (replication factor Y) PriA	`tgtcagtggcctttgcgagaatg`
SCO1966[m4m5m6] SCC54.26c uvrB SC	ABC excision nuclease subunit B excinuclease ABC subunit B (fragment), highly similar to SW:UVRB_BACSU (EMBL:AF017113) Bacillus subtilis excinuclease ABC subunit B (DinA protein) UvrB or DinA or Uvr aa; fasta scores: opt: 2591 Z-score: 2687.6 bits: 507.5 E(): 4.3e-142; 63.667% identity in 600 aa overlap	`tgtcagtgcggggtcgtacggtgg`
SCO2003[m4m5m6] SC7H2.17c polA	DNA polymerase I	`tgtcagtggcacgtggcaggatcg`
SCO3351[m4m5] SCE94.02	putative DNA repair protein, similar to many eg. SW:RADA_ECOLI RadA, DNA repair protein (mechanism unknown) from Escherichia coli (460 aa) fasta scores; opt: 1218, z-score: 1309.3, E(): 0, (41.4% identity in 452 aa overlap)	`tgtcagtgggggcggttacggttcg`
SCO3434[m4m6] SCE36.01 SCE9.41	putative DNA polymerase I hypothetical protein	`tgtcagtcccgcctgcgacactcg`
SCO3541[m4m5m6] SCH5.03c	putative DNA polymerase, len: 401 aa; limited region of similarity to many DNA polymerase III subunits e.g. SW:DP3X_SALTY DNA polymerase III subunits gamma and tau from Salmonella typhimurium (642 aa) fasta scores; opt: 327, z-score: 338.5, E(): 1.5e-11, (32.1% identity in 246 aa overlap)	`tgtcagtgccgccccgcacaatgg`
SCO3540 SCH5.02c slpD	proteinase (putative secreted protein).	
SCO3874[m5] gyrB SCH18.11c	DNA gyrase subunit B	`tgtcagtggtaccggtagaatg`
SCO3873 gyrA SCH18.10c	DNA gyrase subunit A	
SCO5761[m4m5m6] SC7C7.16c	putative ATP-dependent DNA helicase, len: 1690 aa; highly similar to e.g. LHR_ECOLI probable atp-dependent helicase lhr (EC 3.6.1.-) (1538 aa), fasta scores; opt: 2361 z-score: 3018.1 E(): 0, 48.6% identity in 1435 aa overlap	`tgtcagtggccgggtgcacgatgg`
SCO5760	DNA glycosylase involved in DNA repair	

SCO5802[m4m6] SC4H2.23	putative ATP-dependent helicase, probable ATP-dependent helicase, len: 664 aa; similar to e.g. DING_ECOLI P27296 probable ATP-dependent helicase dinG (716 aa), fasta scores opt: 272 z-score: 409.8 E(): 1.3e-15, 26.5% identity in 686 aa overlap	tgtcagtcccgggccgtagggtgg
SCO5815[m4m5m6]	putative ATP-dependent DNA helicase, similar in N-terminal half to many e.g. RECQ_ECOLIP1 5043 atp-dependent dna helicase recq (607 aa), fasta score s; opt: 546 z-score: 615.9 E(): 4.1e-27, 35.9% identity in 362 aa overlap	tgtcagtgccatcgggttgcatgg
SCO5817	putative DNA hydrolase with mutT domain	
SCO5836[m4m5m6] SC9B10.03c	DNA gyrase-like protein	tgtcagtgccgcagtccacaatgg
SCO6084[m4m5m6] SCBAC1A6.08	putative DNA polymerase, len: 244 aa; similar to SW:DP3E_ECOLI (EMBL:X04027) Escherichia coli DNA polymerase III, epsilon chain (EC 2.7.7.7) DnaQ, 243 aa; fasta scores: opt: 137 z-score: 167.9 E(): 0.089; 26.2% identity in 206 aa overlap,	tgtcagtggcccgtgcgagggtgg
2.2.7	Phospholipids (14)	
SCO6445[m4m5m6] SC9B5.12	putative inositol monophosphatase	tgtcagtggcgcgtgctctcatgg
3.2.6	Heme, porphyrin (16)	
SCO4469[m4m5m6] hemL SCD65.12	glutamate-1-semialdehyde 2,1-aminomutase	tgtcagtgacctcccccacggtcg
SCO4470 SCD65.13	putative phosphoglycerate mutase	
SCO5859[m4m5m6] hemH SC9B10.26	ferrochelatase	tgtcagtgccgggtggcaggatcg
4.1.6	Gram +ve membrane (836)	
SCO4069[m4m6] SCD25.05	putative integral membrane protein	tgtcagtgccgggtgccacagtgg
SCO4811[m5] SCD63A.22	putative integral membrane protein	tgtcagtgcggcggactcacg
SCO6538[m6] SC5C7.23	putative integral membrane protein	tgtcagtctcacaggggtgttcg
SCO6794[m4m5] SC1A2.03 cvnA7	putative membrane protein.	tgtcagtgtccgtcgggcgacgtag
SCO7763[m4m5m6] SC5E9.11	putative membrane protein	tgtcagtgcctcctgtcagcatgg
4.2.2	Ribosomal proteins - synthesis, modification (62)	
SCO3906[m4m6] SCH24.28 rpsF	putative 30S ribosomal protein S6	tgtcagtgggtcctggtttactcg
SCO3907 SCH24.29 ssb	putative single-strand DNA-binding protein	

SCO3908 SCH24.30 rpsR<br>SCO3909 SCH24.31 rplI	putative 30S ribosomal protein S18<br>50S ribosomal protein L9	
6.3.2	AraC (33)	
SCO0287^{m6} SCF85.15	putative regulatory protein	tgtcagtggcgcaccgtcactcg
6.3.6	LysR (38)	
SCO3919^{m6} SCQ11.02c abaB	putative lysR-family transcriptional regulator	tgtcagtgtccaatcaccgtag
6.5.0	Others (237)	
SCO2105^{m4m5m6} SC2C1A.01c SC4A10	putative transcriptional regulatory protein (fragment). hypothetical protein	tgtcagtgccaggtggcaggctgg
7.0.0	Not classified (included putative assignments) (565)	
SCO0278^{m5} SCF85.06 MutT<br>SCO3000^{m5} SCE33.02c	domain containing protein<br>putative phosphatase	tgtcagtggcccctgccaggatg<br>tgtcagtggcgccgggtacg
	Not known according to classification	
SCP1.106^{m4m6}<br>SCP1.204c^{m4m5m6}		tgtcagtcgtccgtcctcgcctcg<br>tgtcagtgggggttcgtagggtgg
