# Supplementary material for: SIGffRid: A tool to search for sigma factor binding sites in bacterial genomes using comparative approach and biologically driven statistics
Source: BMC Bioinformatics. 2008 Jan 31;9:73. doi: 10.1186/1471-2105-9-73 (PMC2375139; doi:10.1186/1471-2105-9-73)
Supplement: Additional file 5 — SIGffRid motifs similar to known E. coli, S. typhimurium, B. subtilis, or B. licheniformis SFBSs. SIGffRid results compared with known SFBS motifs in E. coli/S. typhymurium on one hand, and B. subtilis/B. licheniformis on the other hand. Interesting results obtained from E. coli K12/S. typhimurium LT2 and B. subtilis 168/B. licheniformis ATCC 14580 pairs of bacterial genomes by using all pairs of orthologues. [file 1471-2105-9-73-S5.pdf]

Table 6: SIGffRid results compared with known SFBS motifs in *E. coli*/*S. typhymurium* on one hand, and *B. subtilis*/*B. licheniformis* on the other hand

- (dark grey) **σ factor: name [known/deduced consensus if available]**
- (light grey) **known promoters (when no consensus can be clearly deduced)**
- (white) **SIGffRid output motifs**

Motifs are presented as follow: DNA_motif (R ratio, LRT likelyhood ratio test).
(for *E. coli*: $R \geq 0.50$, $LRT \geq 3.84$; for *S. typhimurium*: $R \geq 0.48$, $LRT \geq 3.84$)

SIGffRid results for *Escherichia coli*/*Salmonella typhimurium* pair.

σ **factor:** σ^{70} $ttgacan_{16,18}tataat$ [1]	
**SIGffRid output motifs (spacer range: 14 to 20)**	
E. coli	*S. typhimurium*
$tgnnan_{16,18}tataat$ (0.52, 6.40)	
$tgnnan_{15,18}tataat$ (0.53, 7.18)	
$tn_2cn_{17,19}tataatg$ (0.54, 11.30)	$tn_2cn_{17,19}tataatg$ (0.52, 21.86)
	$tngn_{19,20}tataatg$ (0.49, 12.72)

σ **factor: RpoS** $?n_?ctatact$ [2]*	
**SIGffRid output motifs (spacer range: 14 to 20)**	
E. coli	*S. typhimurium*
$tgn_2an_{17,19}tatact$ (0.73, 10.10)	
$tn_3an_{17,19}tatact$ (0.51, 15.40)	$tn_2an_{17,19}tatact$ (0.49, 41.75)
$tn_3an_{16,18}tatact$ (0.51, 15.43)	
$cn_2gn_{17,19}ctataat$ (0.59, 10.69)	
$tntntn_{18,20}tatact$ (0.55, 6.79)	
$tn_3tn_{16,18}tatactg$ (0.50, 6.36)	$tn_3tn_{16,18}tatactg$ (0.50, 16.79)

*No -35 box is clearly defined.

**Motif putatively recognized by a transcription factor**	
**SIGffRid output motifs (spacer range: 14 to 20)**	
E. coli	*S. typhimurium*
	$tataan_{14,17}ttattt$ (0.66, 13.05, x3)
$tggn_{19,21}tacaat$ (0.53, 9.24)	
$tn_3tn_{14,16}gtatact$ (0.62, 6.84)	$tn_3tn_{14,16}gtatact$ (0.53, 12.70)
$anan_{15,17}tgtat[ag]$ (0.54, 39.94)	
$anan_{14,16}gtataat$ (0.75, 8.17)	
$tngn_{18,19}gtataat$ (0.60, 6.67)	$tnan_{16,18}gtatact$ (0.74, 20.21)
	$tgtgatn_{11,13}ttta$ (0.54, 14.29)

SIGffRid results for *Bacillus subtilis*/*Bacillus licheniformis* pair.

(for *B. subtilis*: $R \geq 0.51$, $LRT \geq 3.84$; for *B. licheniformis*: $R \geq 0.52$, $LRT \geq 3.84$)

σ **factor: SigA** $ttgacan_{16,18}tataat$ [3]	
**SIGffRid output motifs (spacer range: 14 to 20)**	
B. subtilis	*B. licheniformis*
$ttgan_{18,19}tataat$ (0.77, 43.47)	$ttgan_{18,20}tataat$ (0.79, 63.87)
$ttgacn_{18,19}taaaat$ (0.76, 12.85)	$ttgacn_{17,19}taaaat$ (0.66, 11.65)
$ttgacn_{18,20}ataat$ (0.71, 43.15)	$ttgacn_{17,19}tata$ (0.55, 53.42)
$ttn_2cn_{18,19}tataatt$ (0.68, 23.73)	$ttn_2cn_{18,20}tataat$ (0.53, 70.88)
$ttn_2cn_{17,19}tataata$ (0.57, 30.48)	$ttn_2cn_{17,20}tataat$ (0.52, 83.85)
$ttgacn_{17,19}tataa$ (0.67, 29.71)	$ttgacn_{19,21}taata$ (0.68, 22.84)
$ttgacn_{16,18}tataa$ (0.57, 18.10)	$ttgacn_{17,20}tataa$ (0.66, 32.51)
	$ttgacan_{18,19}anant$ (0.53, 42.90)
	$ttgacaan_{17,18}anant$ (0.83, 30.80)
	$ttgacan_{17,18}tan_2a$ (0.58, 41.75)
	$ttgaan_{17,18}tataat$ (0.83, 12.46)
$ttgn_{18,20}ctataat$ (0.67, 28.56)	$ttgn_{18,20}ttataat$ (0.72, 41.17)
	$tn_2an_{16,18}ctataat$ (0.55, 26.28)
	$antn_{18,21}ctataat$ (0.53, 23.48)
$ttgn_{19,20}tacaata$ (0.52, 12.68)	$ttgn_{19,20}tacaata$ (0.81, 19.48)
	$tn_3an_{17,18}tacaata$ (0.52, 12.68)
	$tn_3an_{16,18}tataata$ (0.54, 23.82)
	$tn_3an_{17,19}tataata$ (0.55, 24.40)
$ttgacn_{17,19}ataat$ (0.62, 25.62)	$ttgacn_{17,19}ataat$ (0.60, 22.95)
$gacan_{16,18}tataat$ (0.60, 16.93)	$gacan_{16,19}tataat$ (0.59, 13.63)
	$gacan_{15,18}tataat$ (0.57, 13.37)
$ttgn_{18,20}tataat$ (0.58, 79.09)	$ttgn_{19,21}tataat$ (0.62, 98.65)
	$ttgn_{18,21}tataat$ (0.59, 99.07)
$tnanan_{15,17}tataat$ (0.51, 39.02)	
$tngn_{19,21}tataata$ (0.59, 51.90)	$tngncn_{17,19}tataat$ (0.61, 50.60)
	$tngncn_{17,18}tataat$ (0.74, 48.24)
	$tgncn_{16,18}tataat$ (0.64, 47.86)
$tn_2cn_{18,20}tataata$ (0.57, 30.48)	$tn_2can_{16,18}tataat$ (0.57, 51.72)
$tnan_{17,19}tataat[ga]$ (0.53, 76.73)	$tnan_{16,20}tataatg$ (0.56, 44.52)
$tn_2acn_{17,19}tataat$ (0.51, 47.73)	$tn_2acn_{17,19}tataat$ (0.63, 61.48)
	$tn_2acn_{17,18}tataat$ (0.68, 49.09)
	$ttn_2cn_{18,18}tataatg$ (0.70, 13.88)
	$ttn_2an_{16,19}tataatg$ (0.56, 16.61)
	$ttn_2an_{17,19}tataata$ (0.64, 14.71)
$ttn_2cn_{16,18}tataata$ (0.51, 21.54)	
$ttgacan_{14,15}tn_2ta$ (0.52, 36.05)	
$ttgacan_{16,17}ataa$ (0.57, 10.36)	
$ttn_2tn_{17,19}tataat$ (0.52, 22.78)	

$tgn_2an_{16,18}tataat$ (0.54, 40.24)	$tgn_2an_{16,18}tataat$ (0.53, 30.83)
$tngncn_{17,19}tataata$ (0.75, 24.50)	$tngncn_{17,19}tataata$ (0.76, 26.33)
$ttgcn_{18,20}tataat$ (0.53, 20.08)	$ttgcn_{18,20}tataat$ (0.56, 23.09)
	$tn_2cn_{18,20}tataata$ (0.53, 36.66)
	$tn_2cn_{16,18}ttataata$ (0.65, 21.65)
	$tn_2an_{17,19}ttataat$ (0.52, 34.59)
	$tn_2an_{16,19}tataatt$ (0.52, 30.55)
	$antnan_{18,20}tataat$ (0.54, 21.75)
	$ttgcaan_{16,17}tana$ (0.53, 18.09)
$ttgan_{17,18}ctataa$ (0.54, 11.53)	$ttgan_{18,20}atataa$ (0.53, 9.19)
$tgn_2an_{15,17}atataat$ (0.53, 9.56)	$tgn_2an_{16,18}tataata$ (0.67, 16.36)
$tngn_{18,20}gtataat$ (0.66, 37.73)	$tngn_{18,20}ttataat$ (0.53, 36.81)
	$tngn_{19,21}tataatt$ (0.59, 29.85)
$tgn_2tn_{16,17}tataat$ (0.52, 32.43)	
$antngn_{19,21}tataat$ (0.59, 37.05)	
$ttn_2tn_{17,19}tataata$ (0.52, 22.78)	$ttan_{16,19}tataata$ (0.53, 7.32)
$tntnan_{16,18}ttataat$ (0.61, 23.75)	$tntnan_{17,19}tataat$ (0.57, 39.73)
$tntn_{18,20}tataatag$ (0.76, 27.76)	$tnan_{17,20}tataat[ag]$ (0.58, 74.99)
	$tnan_{16,20}tataata$ (0.58, 40.62)
	$tnan_{16,19}tataatt$ (0.59, 34.69)
	$tnan_{17,19}tataatt$ (0.58, 26.66)
	$tnanan_{15,17}tataat$ (0.59, 34.03)
	$tn_2an_{16,20}tataat[ag]$ (0.53, 73.84)
$gttgacn_{18,20}tan_2a$ (0.59, 40.73)	
$gttgacan_{18,19}an_2at$ (0.54, 11.35)	
	$gtn_2an_{18,20}tataat$ (0.53, 20.13)
$ganan_{17,18}tataat$ (0.63, 24.93)	
	$acntn_{14,16}tataat$ (0.63, 34.78)
	$tan_2gn_{19,21}tataat$ (0.56, 18.90)
	$an_2gn_{18,19}ttataat$ (0.59, 21.30)
$tntn_{16,18}gttataat$ (0.59, 16.73)	$tn_2tn_{16,19}gttataa$ (0.54, 24.08)
	$tn_2tgn_{18,20}tataat$ (0.53, 36.95)
	$tn_2tgn_{19,20}tataat$ (0.55, 32.18)
	$tattgacn_{17,20}anant$ (0.61, 17.11)
	$atn_2an_{18,20}tataat$ (0.54, 28.27)
$attgacan_{17,19}an_2at$ (0.56, 27.34)	$tattgacn_{18,20}an_2at$ (0.62, 19.88)
	$tattgacn_{18,20}anant$ (0.64, 17.88)
	$tattgacan_{18,19}an_3t$ (0.68, 14.96)
	$tattgacan_{19,20}tn_2t$ (0.54, 8.14)

σ **factor: SigW** $tgaaacn_{16,17}cgta$ [4]	
**SIGffRid output motifs (spacer range: 14 to 20)**	
B. subtilis	*B. licheniformis*
$tgaaacn_{16,17}cgtat$ (0.74, 17.71) (found 15 times)	$tgaaacn_{16,17}cgta$ (0.69, 22.09) (found 18 times)

Motif putatively recognized by transcription factors	
**SIGffRid output motifs (spacer range: 14 to 20)**	
B. subtilis	*B. licheniformis*
$attgacan_{13,15}tn_2ta$ (0.60, 21.70)	$attgacan_{14,15}tn_2t$ (0.53, 8.05)
$atttn_{13,15}tataat$ (0.52, 22.36)	$attttn_{12,15}tataat$ (0.58, 16.05)
	$atttn_{13,17}tataat$ (0.54, 29.78)
	$atttn_{13,16}tataat$ (0.57, 29.45)
	$atttn_{12,16}tataat$ (0.54, 29.33)
$tttn_{13,15}tataata$ (0.56, 27.57)	
$tataatan_{12,14}aaa$ (0.56, 11.98)	$tataat[tg]n_{13,17}aaa$ (0.56, 20.28)
	$aaan_{13,17}tataatg$ (0.56, 11.08)
$tataatn_{14,16}ataa$ (0.52, 10.23)	$tataatn_{14,16}ataa$ (0.64, 9.31)
$ttttn_{16,18}tataata$ (0.53, 12.05)	$ttttttn_{12,16}tataat$ (0.55, 8.28)
$cntngn_{19,21}tataat$ (0.54, 31.42)	$cntnan_{15,19}tataat$ (0.67, 52.77)
$gn_2gn_{14,18}tcgtccct$ (0.58, 18.83)	
$gn_2gn_{15,17}tcgtccct$ (0.64, 16.57)	
$gn_2gn_{14,16}tcgtccct$ (0.53, 12.78)	
$ggtn_{17,20}cgtccct$ (0.53, 12.85)	$ggtggn_{13,17}tcgtcc$ (0.69, 19.12)
$an_3tn_{14,19}tatacta$ (0.58, 34.36)	$anantn_{14,17}atacta$ (0.58, 17.57)
	$an_3tn_{13,20}tatacta$ (0.61, 36.86)
$tn_2an_{17,19}tatacta$ (0.56, 22.24)	$tn_2an_{17,19}tatacta$ (0.64, 23.29)
$tntn_{15,17}tatacta$ (0.62, 32.00)	$tn_2tn_{17,20}tatacta$ (0.52, 21.38)
$tngn_{19,21}tatacta$ (0.59, 22.71)	$tngn_{19,21}tatacta$ (0.59, 22.71)
	$anan_{15,18}tatacta$ (0.62, 16.52)
$tatactan_{16,18}aaa$ (0.55, 4.79)	
	$attn_{16,18}atacta$ (0.59, 23.16)
$tttn_{11,13}tatacta$ (0.54, 9.64)	$ttgn_{19,20}tatacta$ (0.88, 16.69)
	$ttgn_{17,20}tatacta$ (0.75, 19.84)
	$acttn_{14,16}tataat$ (0.68, 17.10)
	$cattn_{14,16}tataat$ (0.52, 15.79)

References

[1] Lisser S, Margalit H: **Compilation of *E. coli* mRNA promoter sequences**. *Nucleic Acids Res* 1993, **21**(7):1507–1516.

[2] Becker G, Hengge-Aronis R: **What makes an *Escherichia coli* promoter σ^S dependent? Role of the -13/-14 nucleotide promoter positions and region 2.5 of σ^S**. *Mol Microbiol* 2001, **39**(5):1153–1165.

[3] Wosten M: **Eubacterial sigma-factors**. *FEMS Microbiology Reviews* 1998, **22**:127–150.

[4] Cao M, Kobel P, Morshedi M, Wu M, Paddon C, Helmann J: **Defining the *Bacillus subtilis* σ^W Regulon: A Comparative Analysis of Promoter Consensus Search, Run-off Transcription/Macroarray Anal-

ysis (ROMA), and Transcriptional Profiling Approaches. *J Mol Biol* 2002, **316**:443–457.
